# Bioactive Compounds Isolated from Microalgae in Chronic Inflammation and Cancer

**DOI:** 10.3390/md13106152

**Published:** 2015-09-30

**Authors:** Elena Talero, Sofía García-Mauriño, Javier Ávila-Román, Azahara Rodríguez-Luna, Antonio Alcaide, Virginia Motilva

**Affiliations:** 1 Department of Pharmacology, Faculty of Pharmacy, University of Seville, Seville 41012, Spain; E-Mails: javieravila@us.es (J.A.-R.); arodriguez53@us.es (A.R.-L.); aalcaide@us.es (A.A.); motilva@us.es (V.M.); 2 Department of Plant Biology and Ecology, Faculty of Biology, University of Seville, Seville 41012, Spain; E-Mail: sgarma@us.es

**Keywords:** chronic inflammation, colon cancer, skin cancer, chemoprevention, microalgae

## Abstract

The risk of onset of cancer is influenced by poorly controlled chronic inflammatory processes. Inflammatory diseases related to cancer development include inflammatory bowel disease, which can lead to colon cancer, or actinic keratosis, associated with chronic exposure to ultraviolet light, which can progress to squamous cell carcinoma. Chronic inflammatory states expose these patients to a number of signals with tumorigenic effects, including nuclear factor kappa B (NF-κB) and mitogen-activated protein kinases (MAPK) activation, pro-inflammatory cytokines and prostaglandins release and ROS production. In addition, the participation of inflammasomes, autophagy and sirtuins has been demonstrated in pathological processes such as inflammation and cancer. Chemoprevention consists in the use of drugs, vitamins, or nutritional supplements to reduce the risk of developing or having a recurrence of cancer. Numerous *in vitro* and animal studies have established the potential colon and skin cancer chemopreventive properties of substances from marine environment, including microalgae species and their products (carotenoids, fatty acids, glycolipids, polysaccharides and proteins). This review summarizes the main mechanisms of actions of these compounds in the chemoprevention of these cancers. These actions include suppression of cell proliferation, induction of apoptosis, stimulation of antimetastatic and antiangiogenic responses and increased antioxidant and anti-inflammatory activity.

## 1. Introduction

Cancer constitutes one of the leading causes of death, with an estimated 14.1 million new cases of cancer registerd worldwide in 2012, and it is expected to increase in the coming years [1]. The risk of onset of cancer is influenced by previous chronic inflammatory processes; epidemiological studies have reported that up to 25% of diagnosed tumours present, in their origin or evolution, an important inflammatory component [2]. Chronic inflammation is associated with extrinsic and intrinsic factors that increase the risk of developing cancer; the first consists of microbial infections, such as *Helicobacter pylori* and its association with gastric cancer [3], tobacco and its relation with lung cancer [4] or ultraviolet (UV) light and its association with skin tumors [5]. Intrinsic factors, driven by genetic alterations, include mutations in oncogenes, like RAS oncogenes, in tumor suppressor genes, such as *adenomatosis polyposis coli* (APC) and p53 [6] and in DNA repair genes, such as MSH-2, MSH-6 and PMS-2 [7], which lead to cell transformation and maintain autonomous proliferation of transformed cells. In addition, intrinsic defects comprise alterations of the immune system [8,9]. In some types of cancer, transformed cells produce inflammatory mediators, generating an inflammatory microenvironment in tumors for which there is no underlying inflammatory condition [10].

Until now, cancer research has focused on the search for curative treatments, and few studies have aimed to develop preventive strategies. Chemoprevention is an old concept that consists in the use of drugs, vitamins, or nutritional supplements to reduce the risk of developing or having a recurrence of cancer. Considering the important role of inflammation in the origin and evolution of a variety of tumors, the interest in chemoprevention has markedly increased in the last years [11]. Carcinogenesis of common epithelial tumours, including lung, colon, pancreas, ovary, skin, prostate and breast, which are responsible for most deaths, is a slow process that could start twenty years before the first symptoms appear. This long period is very suitable for using chemopreventive strategies that block the development of invasive and/or metastatic disease. With these objectives, cancer chemoprevention uses natural, synthetic or biological substances to reverse, suppress or prevent either the initial phase of carcinogenesis or the progression of neoplastic cells to cancer [12,13]. Previous studies have indicated that cyclooxygenase 2 (COX-2) inhibitors reduce the risk of colon, lung or skin cancer, and other findings suggest that statins, or certain biguanides such as the classical metformin, are effective chemopreventive agents [14,15,16]. Epidemiological and experimental studies suggest that certain dietary components identified from fruits and vegetables help to maintain a proper balance in cell proliferation and are capable of preventing carcinogenesis. Phytonutrients have received considerable attention due to their low cost and high safety [17]. 

Traditionally, Earth’s natural products have been studied to a great extent for drug development and have been used therapeutically. Cragg *et al.* [18] conducted a detailed study about the importance of natural products as sources of new drugs in the last 25 years. Particularly, they found that 47% of anticancer drugs were of natural origin or directly derived from nature, and up to 70% could be considered structurally related to natural compounds. Early research focused on inland compounds, but in the last 30 years the need for new therapeutic molecules has given rise to a vast number of studies in marine invertebrates and microbes. In this regard, many new interesting compounds, which are commonly referred to as marine natural products, have been discovered. The large diversity found in the marine environment represents a huge source from which to isolate new molecules from microbes such as bacteria, cyanobacteria, fungi, algae, microalgae or small invertebrates [19]. This review summarizes the major bio-products obtained from microalgae (carotenoids, fatty acids, glycolipids, polysaccharides and proteins) with potential interest in the treatment/prevention of inflammatory diseases and colon and skin cancer.

## 2. Microalgae as a Source of Bioactive Molecules

Microalgae are a vast group of unicellular prokaryotic and eukaryotic organisms that are mainly autotrophic, but there are a few taxa such as *Polytoma* sp., *Polytomella* sp., or *Prototheca wickerhamii* that have been described as heterotrophical with degenerated chloroplasts [20,21]. Despite the fact that the number of new species described is increasing yearly, only a small percentage of them have been investigated so far. In fact, only a few phytoplanktonic species are available to grow on a large scale, including *Dunaliella*, *Chlorella*, *Isocrysis*, *Nannochloropsis*, *Nannochloris*, *Chlamydomonas*, *Haematococcus* and *Spirulina*, among others [19,22].

These phytoplanktonic organisms have colonized every type of ecological niche, and they constitute the major group of living organisms in terms of species diversity in both terrestrial and marine waters [23]. Traditionally, the therapeutic compounds have been obtained from inland organisms, but in the last decades the need for new pharmacologic molecules has given rise to a broad number of studies in marine environment in both animals and phototrophic organisms such as microalgae [19,24]. The major interest in microalgae is the capacity to modulate their metabolism according to environmental conditions. Moreover, microalgae are acknowledged to be a diverse source of bioactive molecules that play physiological roles for themselves and their environment. In addition, microalgae have been found to produce new molecular structures due to seawater composition in halogen atoms [25]. Being photoautotrophic, their simple growth requirements and the capacity to modulate their metabolism, make them attractive for demand by the pharmaceutical, food, cosmetic or biodiesel industries. In fact, microalgae are rich in high fatty acids, protein, antioxidant pigments and polysaccharides. In this line, microalgae are also used to feed fish in aquaculture or animal consumption as dietetic supply [26]. 

Microalgae have been shown to produce a huge variety of bioactive products with potential commercial values. Nevertheless, only a few of them, such as β-carotene and astaxanthin, have been produced at industrial scale [27], due to their low production in native microalgae and the difficulty in isolating them by economically feasible means [28,29]. For several years, scientific effort in this area has been undertaken to select high-yield strains, to optimize cultivation or to use genetic engineering to modify the strains to get high-value-added products [30,31,32]. In this context, chemicals as metabolic triggers or enhancers that are able to directly modulate cellular metabolism have been proposed and applied in various commercially viable microalgae [33]. In this regard, Franz *et al.* [34] demonstrated that several chemicals such as epigallocatechin gallate, cyclin-dependent kinase 2 inhibitor and cycloheximide could act as enhancers to produce an increase of intracellular lipids in several species of *Nannochloropsis* and *Phaeodactylum tricornutum*. On the other hand, genetic modification is also a helpful tool to obtain a high-yield production of bioactive compounds. In this line, a short exposure to UVC radiation led to a significant increase in total cellular lipid content, including eicosapentaenoic acid (EPA), in *Nannochloropsis* sp., microalgae identified as a highly efficient producer of *n*-3 fatty acids [35]. Moreover, the use of auxins has been shown to regulate growth and metabolite production of several microalgae, such as *Chorella vulgari* [36]. Thus, more studies are required to establish the optimal culture conditions to obtain high concentrations of bioproducts. Many of these biomolecules such as fatty acids, carotenoids, proteins, polysaccharides, phenolic compounds, amongst others, have attracted the interest of the pharmaceutical industry based on their anti-oxidant, anti-inflammatory or anticarcinogenic activities, which make them very promising tools for the prevention of inflammatory diseases or cancer (Table 1). 

**Table 1:** Compounds obtained from microalgae and their biological activities.

Compound	Source	Activity	References
CAROTENOIDS			
β-Carotene	*Dunaliella salina Haematococcus* sp.	Antioxidant Pro-vitamin A Anti-inflammatory Anticancer	[30,37,38]
Astaxanthin	*Haematococcus pluvialis Chlorella zofigiensis Chlorococcum* sp.	Antioxidant Anti-inflammatory Anticancer	[39,40]
Lutein	*Dunaliella salina Chlorella sorokiniana Chlorella prothecoides*	Antioxidant Anti-inflammatory Anticancer	[41,42,43]
Violaxanthin	*Dunaliella tertiolecta Chlorella ellipsoidea*	Anti-inflammatory Anticancer	[44,45]
Zeaxanthin	*Synechocystis* sp. *Chlorella saccharophila*	Antioxidant Anti-inflammatory	[46,47]
Fucoxanthin	*Phaeodactylum tricornutum Isochrysis* sp*.*	Anticancer	[48,49]
FATTY ACIDS			
Eicosapentaenoic acid (EPA)	*Tetraselmis* sp.	Antiinflammatory Anti-angiogenic	[50,51]
Docosahexaenic acid (DHA)	*Tetraselmis* sp*.*	Antiinflammatory Anti-angiogenic	[52,53]
Docosapentaenoic acid (DPA)	Nannochloropsis oculata	Antiinflammatory	[52,54]
GLYCOLIPIDS			
Monogalactosyldiacylglycerol (MGDG)	*Gymnodinium mikimotoi Stephanodiscus* sp *Pavlova lutheri Stephanodiscus* sp.	Anticancer Antioxidant	[55,56]
Digalactosyldiacylglycerol (DGDG)	*Stephanodiscus* sp	Anticancer Antioxidant	[57,58]
Sulfo-quinovosyl-acyl-glycerol (SQAG)	*Stephanodiscus* sp	Anticancer Antioxidant	[57,59]
POLYSACCHARIDES			
Sulphated extracellular polysaccharide	Diatom *Phaeodactylum tricornutum*	Anti-inflammatory Inmunomodulating	[60]
Sulphated polysaccharide Β-(1,3)-glucan	Chlorophyte *Chlorella stigmatophora Chlorella vulgaris*	Anti-inflammatory Inmunomodulating Anticancer	[60,61]
Sulphated polysaccharide	Prasinophyte *Tetraselmis suecica*	Anti-inflammatory	[62]
Sulphated polysaccharide	Haptophyte *Isochrysis galbana*	Anticancer	[63]
Sulphated polysaccharide	Rhodophyte *Porphydium* sp.	Anti-inflammatory Inmunomodulating Anticancer	[64]
Sulphated polysaccharide	Dinoflagellate *Gyrodinium impudicum*	Anti-inflammatory Inmunomodulating Anticancer	[65]
Extracellular polysaccharide s-Spirulan	Cyanobacteria *Arthrospira platensis*	Anticancer	[66]
PROTEIN AND PEPTIDES			
Phycobiliproteins	*Spirulina platensisPorphyridium* sp.	Antioxidant Anti-inflammatory Anticancer	[67,68]
Peptides	*Chlorella pyrenoidosa* Cyanobacteria	Antioxidant Anti-inflammatory Anticancer	[69,70]
OTHER COMPOUNDS			
Amides	*Lyngbya majuscule*	Anticancer	[71]
Quinones	*Calothrix* sp.	Anticancer	[72]
Phenolic compounds	*Spirulina maxima Chlorella ellipsoidea Nannochloropsis* sp	Antioxidant	[73,74]
Tocopherols	*Porphydium* sp.	Antioxidant	[75]

## 3. Colorectal Cancer as a Consequence of Chronic Inflammatory Disorder

### 3.1. Molecular Pathways of Colon Carcinogenesis

Colorectal cancer (CRC) is the third most common cancer, with an estimate average incidence of 42.4 per 100,000 men and women per year, and the third leading cause of death related to cancer disease, being diagnosed each year in about 1 million people worldwide [76]. Inflammatory intestinal conditions implicated in the origin of neoplasia have been the object of numerous clinical, genetic and molecular studies in humans and experimental animal models. Although the association between ulcerative colitis (UC) and elevated risk for CRC is clear, there have been some debates about whether Crohn’s disease (CD) possesses a similar risk [77]. Many papers have reported that the duration of the disease, its severity, the association with other inflammatory diseases including sclerosant colangitis, or certain treatments, are factors that concede inflammation an advantageous role in colon carcinogenesis [78].

Experimental animal models of inflammatory bowel disease (IBD) have been widely tested, but only a few are applicable in the study of the inflammation and associated cancer [79,80]. The histological changes in IBD patients who develop neoplasms correspond to the inflammation-dysplasia-cancer sequence. In this progression, the histological classification has been established by Riddle [81] and Pascal [82]: (i) Undefined for dysplasia/probably negative; (ii) Undefined for dysplasia/probably positive; (iii) Low grade dysplasia; (iv) High grade dysplasia; (v) Carcinoma. However, the identification of dysplasia in intestinal inflammatory diseases represents a huge challenge for both clinicians and pathologists, so a clear diagnosis of dysplasia in IBD is not always possible. Possible markers, such as p53 and alpha-methylacyl coenzyme, have been used. The combination of these two markers is positive in 75.8% for cancers and 30.3% for undefined biopsies for dysplasia, while it is only positive in 0.6% for non-neoplastic epithelium. The suspect lesions can be visible macroscopically or only microscopically. Bird and Good [83] described focal points of aberrant crypts such as preneoplastic lesions in rodents treated with carcinogen. It has been proved that these aberrant crypts can be high or flat lesions; high crypts are raised above the surrounding epithelium with round, elongated, open lights; and flat crypts are small or slightly enlarged lesions with compressed open lights. β-catenin expression has been studied in both high and flat aberrant crypts; this protein is found in the cytoplasm in flat lesions, and it moves to the cytoplasm and nucleus in polypoidal lesions, being an important early event in the development of colitis-associated cancer [84]. 

The molecular mechanisms to explain how a chronic inflammatory state affects tumor development are beginning to be elucidated. These mechanisms comprise the production of inflammatory mediators such as chemokines and cytokines, including tumor necrosis factor alpha (TNF-α), interleukin 1 (IL-1), IL-6, IL-10, IL-11, IL-17, IL-18, IL-22 and IL-23, prostaglandins (PGs) produced by the coordinated enzymatic activity of COX and membrane-associated PGE synthase-1, nitric oxide (NO) released by inducible nitric oxide synthase (iNOS), or signaling pathways including signal transducer and activator of transcription 3 (STAT3) and nuclear factor kappa B (NF-κB). In this regard, NF-κB has been shown to have a role in uncontrolled cell growth, inhibition of apoptosis and induction of metastasis and angiogenesis [85]. Pharmacological strategies for its regulation have been designed, and some of them are directed towards the control of the mechanisms that trigger a persistent activation, as occurs in inflammatory pathologies.

Regarding PGs, these molecules have been found in different types of tumors, including colon adenocarcinomas [86] or squamous cell carcinomas [87], and have tumorigenic effects, including stimulation of cell growth and angiogenesis, inhibition of apoptosis and suppression of immune system. NO radical has a complex behaviour depending on the synthesizing enzyme, levels produced and the tissue microenvironment. One of the main mechanisms by which NO regulates the function of the target proteins is through the S-nitrosation of thiols, which involves the conversion of thiol groups in proteins to form S-nitrosothiols. This is a mechanism for post-translational regulation of protein, but in cancer the consequences of S-nitrosylation are not fully understood and present dual functions: protective effect by inducing cell death or metastasis and epigenetic changes [88]. In addition to NO radicals, reactive oxygen species (ROS) are formed during the oxygen mitochondrial metabolism and have important roles in cell signaling and homeostasis; however, under stress circumstances ROS levels can increase dramatically, resulting in severe damage in cell structures. [89].

**Figure 1:**
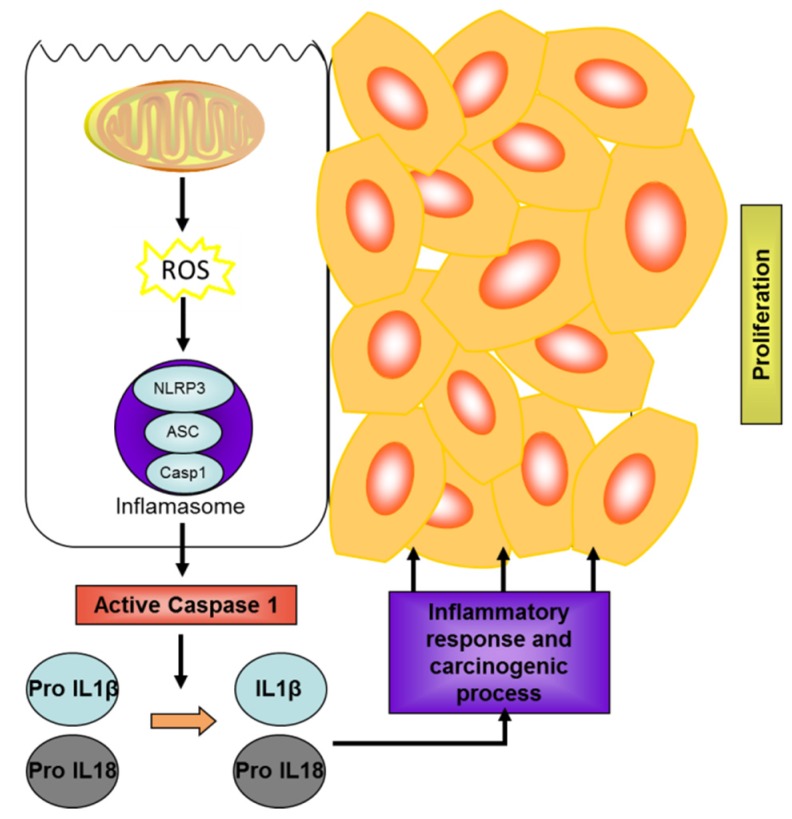
Inflammasome activation and role in carcinogenesis. Reactive oxygen species (ROS) liberated by mitochondria initiate inflammasome assembly, which promotes the maturation of the inflammatory cytokines (IL-1β and IL-18) through caspase 1 activation with the participation of multiprotein oligomers, as the scheme of NLRP3 subtype shows.A microenvironment of chronic inflammation promotes cancer development.

The innate immune system, in response to harmful stimuli, such as pathogens, dead cells or irritants, starts defence programs to repair damaged tissue. Insufficient inflammation can result in persistent infection by pathogens, while excessive inflammation can cause chronic inflammatory pathologies, including arthritis, diabetes, inflammatory bowel disease, or skin diseases [90]. The innate immune function depends upon the recognition of pathogen-associated molecular patterns (PAMPs), derived from invading pathogens, and damage-associated molecular patterns (DAMPs), induced as a result of endogenous stress, by pattern-recognition receptors (PRRs). Nucleotide binding and oligomerization domain (NOD)-like receptors (NLRs), included in the family of PRRs, are found in the cytoplam and are essential for detecting invading pathogens and initiating the innate immune response [91]. Some types of these receptors form a multiprotein complex called inflammasome, with the NLRP3 inflammasome being the best characterized. NLRP3 can be activated by a wide range of stimuli, including pathogens and their components, chemical irritants and endogenous danger signals such as adenosine triphosphate (ATP) [92]. NLRP3 activation is also induced by the production of ROS, whose predominant source generated by danger signals is most probably the mitochondria [93]. Upon stimulation, NLRP3 associates with the adaptor protein ASC, and induces caspase-1 activation and subsequent proteolytic maturation of pro-inflammatory cytokines IL-1β and IL-18 (Figure 1) into their mature active forms [94].

Recent studies have shown that inflammasomes regulate the gastrointestinal (GI) microbiome and can thereby affect host susceptibility to diseases beyond the GI tract, including obesity and diabetes [95]. Inflammasomes also maintain the integrity of the intestinal epithelium and promote its repair, being important factors in the pathogenesis of IBD. In this regards, it has been observed that polymorphisms in genes that encode IL-18 and IL-18 receptor accessory protein are associated with increased susceptibility to CD [96]. Given its association with a number of inflammatory diseases, NRLP3 type inflammasome has been of great interest; polymorphisms in this inflammasome, which impaired IL-1β production by lipopolysaccharide (LPS)-stimulated monocytes, were linked to increased susceptibility to CD. Chen and Nuñez [97] have recently revised complementary information on the role of the inflammasome in IBD.

In pathological conditions, mitochondrial malfunctions induce the activation of NLRP3 inflammasome, and some inflammatory diseases are associated with defects in these organelles and their elimination by autophagy (mitophagy). In IBD pathogenesis, particularly in CD, a a key role of IL-1β, IL-18 and the inflammasome has been reported [98]. In animal models of IBD, genetic defects in autophagy induce hyperactivation of the inflammasome, and the application of anti IL-1β and IL-18 in animals with colitis by dextran sulfate sodium (DSS) reduce colon damage [99]. These observations are consistent with other studies showing that the silencing of genes that regulate autophagy, including Beclin 1, ATG5, or microtubule-associated protein light chain 3 (LC3), cause spontaneous NLRP3 activation and subsequent secretion of IL-1β [100].

In IBD-associated colorectal cancer, the activation of the inflammasome is a consequence of the inflammatory pathology and plays a key role in tumorigenesis. In animals IL18^−/−^ or deficient in its receptor, the combination of azoxymethane (AOM) and DSS showed a large number of tumors, and the authors suggested a role of NLRP3 in the supression of such tumors [101]. Similarly, Zaki *et al.* [98] have demonstrated that caspase-1-deficient mice have increased the number of tumors, similar to NLRP3 mutants, which is associated with a decreased production of IL-18 and activation of STAT1. NLRP6 is expressed particularly in the hematopoietic system, and similar to NLRP3, could protect against the development of tumors; this result is remarkable because of the high expression of NLRP6 in the intestinal epithelium. Regarding the NLRC4 inflammasome, findings are contradictory, with some studies showing negative regulatory effects [102] and others reporting no significant effect [103]. A recent retrospective analysis of gene expression from human patients [104] to elucidate the contribution of NLR family members in IBD and cancer revealed that NLRP1 was significantly dysregulated; complementary data in Nlrp1b deficient mice confirmed that NLRP1 attenuates GI inflammation and tumorigenesis. Both results identify NLRP1 as an essential mediator of the host immune response during intestinal inflammation and cancer.

Sirtuins are a group of highly phylogenetically conserved proteins that catalyze the deacetilation of target proteins, in a nicotinamide adenine dinucleotide (NAD^+^)-dependent manner [105]. In mammals, there are seven sirtuins with diverse cellular locations that regulate a variety of physiopathological processes, such as aging, inflammation and cancer, through deacetylation of key transcription factors, enzymes and proteins [106]. Sirtuin 1 (SIRT1), the most studied mammalian homologue, has demonstrated its relation with inflammation through modulation of the activity of NF-κB [107]. More recently, SIRT6 has been shown to be implicated in DNA repair, telomere maintenance, glucose homeostasis, inhibition of obesity induced by metabolic dysfunction and attenuation of inflammation [107,108].

Autophagy is a biological process involved in recycling long-lived proteins and damaged organelles, including mitochondria, to maintain cellular homeostasis. This process is initiated by the formation of double-membrane bound vacuoles, the autophagosomes, which envelop cytoplasmic contents and deliver them to autolysosomes [109]. Autophagy is regulated by nutrient-sensing pathways, including the mammalian target of rapamycin (mTOR), AMP-activated protein kinase (AMPK) and SIRT1. AMPK is a major metabolic energy sensor, which is activated by a decreased ATP/AMP ratio, such as occurs during nutrient starvation and hypoxia [110]. Accumulating evidence indicates that SIRT1 and AMPK mutually regulate each other and both are required for autophagy induction (reviewed in Garcia-Mauriño *et al.* [111]). 

Autophagy is suggested to be involved in the regulation of IBD and CRC. Autophagy malfunction is associated with an alteration of both innate and adaptative immune responses, defects in bacterial clearance and dysfunction of goblet and Paneth cells; all these perturbations are related to IBD and CRC pathogenesis [111]. It has been reported that the initial activation of this process may have a beneficial role in disease, since it diminishes the risk of damage by elimination of anomalous proteins or injured organelles. It has been reported that mice lacking essential components of the autophagic machinery, such as the autophagy-related 16-like 1 (Atg16L1) are highly susceptible to colitis [99]. As regards to cancer, it is not clear whether autophagy suppresses or facilitates tumor development. As a tumor suppressing mechanism, autophagy serves as an alternative to apoptosis to eliminate transformed cells. However, it has also been reported that autophagy may facilitate cancer cells growth and survival under stressful conditions such as nutrient starvation and low-oxygen conditions, especially in the inner parts of the tumors [112]. This process is suggested to be mediated by AMPK, which is activated as a consequence of decreased intracellular ATP level, as occurs under nutrient deprivation or hypoxia. In this situation, AMPK-mediated induction of autophagy could be advantageous for the tumor development by allowing the tumor cells to cope with the stress [113]. On the other hand, AMPK, in addition to activating autophagy, has shown cytotoxic activity in several cancer cell lines; this supports the fact that AMPK is a tumor suppressor, and thus a potential therapeutic target. In this regard, metformin, an antidiabetic drug that activates AMPK, reduces the incidence of tumors including CRC in treated patients [114].

A role of SIRT1 in tumorigenesis is still controversial because this protein has been shown to act as both tumor suppressor and tumor promoter. In this line, it has been reported that SIRT1 overexpression in the intestine reduces polyp formation, a potential precursor to CRC, in Apc^min/+^ mice, possibly through deacetylation of β-catenin [115]. However, SIRT1 deficiency led to increased tumor formation in p53-null mice [116] and in a mouse xenograft model of colon carcinoma [117]. Moreover, a previous paper demonstrated that SIRT1 expression correlated with stage of different human CRC, detecting the lowest SIRT levels in advanced adenocarcinomas and metastatic tissue samples [117,118]. However, the results of other investigations have reported a protumoral activity of SIRT1 in CRC. In this line, a recent study has demonstrated an increased SIRT1 expression in different colonic lesions, including polyps, adenomas and neoplasia, which was associated with the grade of malignancy and invasiveness [119]. Further in line with tumor-promotive function, SIRT1 stimulated constitutive Wnt signaling and Wnt-induced cell migration in the colon cancer cell lines HT-29, HCT116, RKO and DLD-1 [120]. SIRT6 has shown several catalytic activities including deacetylation, deacylation and ribosylation. This protein has been recently categorized as a tumor suppressor because its depletion alters the levels of acetylated histone H3. The deacylation activity has been endorsed to the regulation of TNF-α secretion. SIRT6 also undergoes auto-ribosylation that might deliver to a self-regulation of catalytic functions, and also enhances poly (ADP-ribose) polymerase 1 (PARP1)-dependent DNA repair under oxidative stress and aging. Recent studies evaluated that SIRT6 deficiency is associated with various diseases including inflammation and different types of cancer [121]. These data suggest that SIRT6 may be a promising target for cancer prevention and therapy.

### 3.2. Microalgae as a Source of Biomolecules with Potential in IBD and Colon Cancer

#### 3.2.1. Carotenoids

Carotenoids are natural isoprenoid pigments biosynthesized by all photosynthetic plants, protists, and bacteria, as well as some heterotrophic bacteria, some fungi and some invertebrates. Animals are generally unable to synthesize carotenoids and require a dietary intake to meet daily health demands. The main subtypes of carotenoids are carotenes (hydrocarbon carotenoids) and xanthophylls (oxygenate derivatives). Carotenoids comprise many of the yellow, orange and red pigments of nature, including many fruits, vegetables, flowers, butterflies and crayfish [122]. Five major carotenoids are synthetically produced on an industrial scale (lycopene, β-carotene, canthaxanthin, zeaxanthin and astaxanthin) for use in a range of food products and cosmetics, such as vitamin supplements and health products and as feed additives for poultry, livestock, fish and crustaceans [30].

Microalgae are a rich source of carotenoids [123,124,125]. Marine microalgae contain up to 0.2% of carotenoids. For this reason, they are not only a valuable source of the purified compounds, but also potential functional foods that are at present being studied as chemopreventive agents against inflammation and cancer [44,126].

The main sources of carotenoids are microalgae that belong to the Chlorophyceae family. *Dunaliella* has the highest content of β-carotene (up to 1% dry weight), and *Haematococcus pluvialis* accumulates the highest levels of xanthophylls (astaxanthin). Microlgae synthesize all xanthophylls produced by higher plants (violaxanthin, anteraxanthin, zeaxanthin, neoxanthin and lutein), and they can also produce others, such as astaxanthin, loroxanthin and caraxanthin [123]. Fucoxanthin, diatoxanthin and diadinoxanthin are produced by brown algae or diatoms [127]. 

##### 3.2.1.1. β-Carotene

The main microalgae used as source of β-carotene (β,β-carotene) are *Dunaliella* and *Haematococcus* [38]. *Dunaliella salina* is grown on large outdoor terrains in some countries like Israel, producing large amounts of β-carotene for the market [30]. The biomass of some marine *Tetraselmis* and *Pyramimonas* strains is also used for fish food additives [128].

Fruits and vegetables are the main sources for human dietary carotenoids. β-Carotene is the most prominent member of the group of carotenoids that is present in the human diet, and it is an important source of vitamin A [129]. Some of the antioxidant and anticancer effects of β-carotene are related to its processing to retinol. The retinoids are a class of natural and synthetic molecules structurally related to vitamin A [130]. These compounds have a broad spectrum of biological activities and can influence reproduction, embryogenesis, growth, differentiation, proliferation, apoptosis, vision, bone formation, metabolism, hematopoiesis and immunological processes [131]. Vitamin A and retinoids can influence oncogenesis and prevent several types of cancer [132]. The β-carotene rich marine microalga *Dunaliella bardawil* has been used as source of retinol in a rat diet [133]. Both dried *Dunaliella bardawil* and an oil extract of the alga were shown to satisfy the total requirement of retinol in rats.

The anti-inflammatory activity of β-carotene has been shown in many *in vitro* and *in vivo* models [134,135], and it is applicable in IBD. Although β-carotene was reported to have no effect on inflammatory markers, it altered their proteomic response in Caco-2 intestinal epithelial cells and could act preventively on intestinal inflammatory diseases such as CD and UC [136]. Recently, it was shown that β-carotene treatment ameliorated the severity of UC in a DSS mouse model [137]. The effect was related to modulation of various molecular targets, such as NF-κB, COX-2, matrix metalloproteinase 9 (MMP-9), among others, and it involved a decrease of both local and systemic damage. *Dunaliella bardawil* was used to pre-feed rats, and this treatment protected against acetic-acid induced bowel inflammation [138]. It is worth noting that patients affected by early-stage IBD had low serum concentrations of micronutrients including Se and β-carotene [139]. Similarly low levels of β-carotene were found in CD [140,141] and in UC and CD subjects [142,143]. These results suggest that UC and CD patients could benefit from the consumption of natural *Dunaliella*-derived β-carotene.

Several *in vitro* studies showed that β-carotene displayed growth inhibitory and pro-apoctotic effects on human colon cancer cell lines [144]. Animal models of colon carcinogenesis have shown that dietary supplementation of β-carotene had anticancer effects on AOM-induced colon carcinogenesis in rats [145]. In addition, both β-carotene and retinoic acid decreased migration, invasion and MMP expression in LoVo colon carcinoma cells [146]. MMPs are required for invasion of tumor cells into a new tissue. Despite the prevailing experimental evidence for a beneficial role of β-carotene, there are some controversial results, such a study with male F344 rats that investigated the role of β-carotene and lutein in CRC [147]. Data showed that the chemopreventive activity of these compounds against colon carcinogenesis depended on the dose level, with the highest dose even being harmful.

Consumption of β-carotene has been inversely correlated with CRC risk. Epidemiologic studies indicate that an increased intake of fruits and vegetables that contain carotenoids, such as β-carotene and retinol, is associated with a decreased risk of many types of cancer and degenerative diseases probably due to their antioxidant and anti-inflammatory activities [148]. This association was not found for carotenoids in some studies. For example, in a pooled analysis of 11 cohort studies (North America and Europe) the data did not suggest that dietary carotenoids played an important role in the etiology of CRC [149]. The Fukoaka CRC study in Japan showed that retinol intake was inversely associated with cancer risk, but this association was not shown for dietary β-carotene [150]. The association of prediagnostic plasma concentration and dietary comsumption of carotenoids and vitamins A, C and D with the risk of CRC was examined in a case-control study nested within the European Prospective Investigation into Cancer and Nutrition study [151]. An association between higher prediagnostic plasma retinol concentration and lower risk of CRC was found. Although results also showed an inverse association for dietary β-carotene and vitamins A, C and D with CRC, this was not found for plasma concentrations, suggesting that the possible inverse association between fruit and vegetable consumption and CRC may therefore not be based on these compounds. On the other hand, the findings that several retinoic acid metabolizing enzymes are significantly overexpressed in CRC [152], and that enzymes of the retinoid acid biosynthetic pathway are dysregulated [153], highlight the importance of retinoic acid in CRC.

Nevertheless, an attenuation of the CRC risk was associated with increased dietary β-carotene in many other studies. A study in Israel showed an inverse association of most carotenoids, including β-carotene, lutein and zeaxanthin, with CRC risk. Smoking attenuated this protective effect [154]. In another study, a relatively high serum level of β-carotene was inversely associated with the risk of CRC in postmenopausal women [155]. Similarly, a study in a cohort of male health professionals in the USA found that a diet high in carotenoids was associated with a reduced risk of colorectal adenomas (inverse associations for β-carotene and lutein/zeaxanthin) [156]. Most recently, a study of the association between dietary carotenoid intake and CRC risk in Chinese adults showed inverse associations between β-carotene intake and CRC risk only in males [157].

In summary, there is an important potential for *Dunaliella* biomass or *Dunaliella*-derived β-carotene in UC and CD. It also seems that β-carotene may be important as source of retinol in CRC, although more information relative to retinoic acid metabolism is necessary.

##### 3.2.1.2. Astaxanthin

The ketocarotenoid astaxanthin (3,3′-dihydroxy-β,β-carotene-4,4′-dione) is a red pigment common to many marine animals contributing to the reddish pink color of their flesh [158]. The main sources of astaxanthin are *Haematococcus pluvialis*, *Chlorella zofigiensis* and *Chlorococcum* sp. [39,40]. The amounts collected by the green alga *Haematococcus pluvialis* exceed any other reported source, corresponding to up to 4%–5% of dry weight. The use of *Chlorella zofigiensis* as an alternative to *Haematococcus pluvialis* for production of astaxanthin is reviewed in Liu *et al.* [40]. Mammals lack the ability to synthesize astaxanthin or to convert dietary astaxanthin into vitamin A; unlike β-carotene, astaxanthin has no pro-vitamin A activity [159].

There has been growing interest in the use of astaxanthin as a food-coloring agent, natural feed additive for the poultry industry and for aquaculture, especially as a pigment for fish feeds in the culture of salmon and trout. There have also been reports concerning its application in medicine due to its potent bioactivities including its antioxidative, anticancer, antidiabetic and anti-inflammatory activities, gastro-, hepato-, neuro-, cardiovascular, ocular and skin-protective effects and other actions [39,160].

Asthaxanthin has been used for preventing inflammatory processes, such as colitis, and some types of cancer, including CRC, in cellular and animal models. The anti-inflammatory activity of astaxanthin in cellular models is due to supression of iNOS expression [161]. The inhibitory effect of astaxanthin on the production of NO has important implications for the treatment of IBD.

Astaxanthin could inhibit the growth of human CRC cells, including HCT-116, HT-29, LS-174, WiDr and SW-480 [126]. A significant decrease in the incidence of induced CRC in rats fed with astaxanthin *versus* animals administered only the carcinogen was found [162]. The inhibitory effect of astaxanthin against chemically induced colonic pre-neoplasic progression has also been demonstrated in a dimethylhydrazine (DMH)-induced rat colon carcinogenesis model [163]. In the same line, astaxanthin induced apoptosis in DMH-induced rat colon carcinogenesis through the regulated expressions of COX-2, NF-κB, MMP-2 and 9, proliferating cell nuclear antigen (PCNA) and extracellular signal-regulated kinase (ERK-2) [164]. Dietary astaxanthin ameliorated the colonic inflammation induced by DSS in mice by reducing the proinflammatory factors TNF-α, IL-1β, COX-2 and NF-κB [165]. In the same study, it was shown to reduce AOM/DSS-induced colorectal carcinogenesis, partly due to its anti-inflammatory effects as well as to suppression of proliferation and induction of apoptosis. 

There are no clinic trials with astaxanthin and IBD or CRC patients. Nevertheless, several studies in humans show beneficial effects for astaxanthin related to its antioxidant activity, such as prophylaxis/regression of stomach ulcers caused by *Helicobacter pylorii* infection [166]. In the same line, astaxanthin enhanced both cell-mediated and humoral immune responses in young healthy females, in a randomized double-blind, placebo-controlled study [167], and it also improved facial elasticity in a model of proaged skin in humans [168]. These studies did not reveal any harmful effect.

##### 3.2.1.3. Lutein

Lutein (3*R*,3′*R*,6′*R*-βε-carotene-3,3′-diol) is a yellow oxycarotenoid or xanthophyll containing two cyclic end groups (one beta and one epsilon-ionone ring). In green microalgae, lutein protects cells from ROS damage under stress conditions. Lutein has been widely used as a feed additive and a food coloration agent in industry [169], and it may also protect against age-related macular degeneration in humans [170]. The role of the dietary xanthophyll carotenoids, lutein and zeaxanthin has been extensively studied in adult onset macular degeneration. At present, lutein is mainly produced from the flowers of marigold, but the content is low, about 0.3% dry biomass [171]. This has led to considerable interest in other sources of lutein, notably microalgae, as they have a high lutein content (0.5%–1.2% dry weight) [169]. A *Dunaliella salina* strain developed for β-carotene production has been shown to be a potential producer of lutein under environmental stress conditions [41]. *Chlorella sorokiniana* is also a good source for lutein, and the yield can be improved by managing several nutritional and environmental factors and by random mutagenesis [44]. *Chlorella prothecoides* has also been proposed as a potential source of lutein [43]. 

Many of the beneficial effects of lutein are linked to its ability to quench singlet oxygen, and it is also an excellent free radical scavenger [172]. This antioxidant activity is thought to be responsible for reducing injury due to oxidative and inflammatory processes in cells and tissues [48,173]. An *in vitro* study showed a cytoprotective effect of lutein on the human colon adenocarcinoma cell line HT-29 against deoxynivalenol-induced oxidative stress and inflammation, by maintenance of glutathione levels, inhibition of nuclear migration of NF-κB, downregulation of COX-2 expression and prevention of apoptosis [174].

Lutein also has anticancer activity [175]. As previously mentioned for β-carotene, a study with male F344 showed that the chemopreventive activity of both carotenoid compounds against colon carcinogenesis was dependent on the doses [147]. Extracts from *Chlorella vulgaris*, whose main constituent was lutein, displayed anti-proliferative effects on a human colon cancer cell line (HCT-116) [176]. More recently, lutein showed chemoprotective activity against CRC induced by DMH in the rat by modulating the proliferative activity of K-ras, protein kinase B (PKB) and β-catenin [177].

##### 3.2.1.4. Violaxanthin

Violaxanthin (5,6,5′,6′-diepoxy-5,6,5′,6′-tetrahydro-β,β-carotene-3,3′-diol) is a natural xanthophyll pigment with an orange color found in a variety of plants, macro- and micro-algae. This product has been isolated from several microalgae including *Dunaliella tertiolecta* [44] and *Chlorella ellipsoidea* [45]. 

Violaxanthin had antiproliferative and pro-apoptotic activity against human cancer cell lines [44]. Extracts from the marine microalga *Chlorella ellipsoidea*, whose main constituent was violaxanthin, displayed anti-proliferative effects on a human colon cancer cell line (HCT-116) by inducing apoptosis [176]. In addition, violaxanthin isolated from *Chlorella ellipsoidea* showed antiinflamatory activity when it was tested on LPS-stimulated RAW 264.7 mouse macrophages, by inhibiting NF-κB activation and NO and prostaglandin E2 (PGE2) production [45]. The anti-inflammatory and anticancer activities of violaxanthin may be of interest in IBD and CRC.

##### 3.2.1.5. Zeaxanthin

Zeaxanthin (β,β-carotene-3,3′-diol) together with lutein accumulate in the central retina. Epidemiologic studies suggest that insufficient dietary lutein and zeaxanthin intake or lower serum zeaxanthin levels are associated with increased risk for age-related macular degeneration [178]. Zeaxanthin has been obtained from the cyanobacterium *Synechocystis* sp. and *Microcystis aeruginosa* [46,179], *Nannochloropsis oculata* [180] and *Chlorella saccharophila* [47].

Similarly to lutein, zeaxanthin showed antioxidant and anti-inflammatory properties [181]. Most studies were related to the protective effect of lutein and zeaxanthin against the development of macular degeneration.

A study investigating the association between serum concentrations of carotenoids and the presence of colorectal polyps and cancers in Japanese people showed that high serum zeaxanthin was associated in males with decreased rates of polyps and cancer, and in females, with cancer development [182].

##### 3.2.1.6. Fucoxanthin

The xanthophyll fucoxanthin [(3S,3′S,5R,5′R,6S,6′R,8′R)-3,5′-dihydroxy-8-oxo-6′,7′-didehydro-5,5′,6,6′,7,8-hexahydro-5,6-epoxy-β,β-caroten-3′-yl acetate] is a marine carotenoid found in numerous classes of microalgae (bacillariophytes, bolidophytes, chrysophytes, silicoflagellates, pinguiophytes) and brown macroalgae (phaeophytes) [183,184]. Diatoms exhibit a characteristic golden-brown color due to a high amount of the xanthophyll fucoxanthin that plays a major role in the light-harvesting complex of photosystems. The diatom *Phaeodactylum tricornutum* [48] has been proposed as a commercial source for fucoxanthin, with a production higher than 1.5% dry weight. A similar amount of fucoxanthin was produced by the Haptophyta *Isochrysis* sp. [49]. 

The increasing interest for this carotenoid is mainly due to its anti-obesity effect, primarily detected by murine studies, which showed that fucoxanthin induced the expression of uncoupling protein-1, thus promoting the oxidation of fatty acids and heat production [185]. In addition, fucoxanthin has shown a great antioxidant activity, anti-cancer, anti-diabetic and anti-photoaging properties [183].

Fucoxanthin displayed anticancer activity in several experimental models against a variety of cancer types [186]. With respect to CRC, fucoxanthin has shown cytotoxic activity against several human colon cancer cell lines [187,188], inducing apoptosis and cell cycle arrest. The effect of fucoxanthin on cell viability of colon cancer cell lines (Caco-2, HT-29, and DLD-1) was higher than the effect of other carotenoids, such as β-carotene and astaxanthin [187].

#### 3.2.2. Fatty Acids

Over the last decades, microalgal lipids have gained significant importance, not only due to their feedstock for biofuels production, but also as important biological molecules for the treatment of inflammatory pathologies such as IBD, atherosclerosis, Parkinson’s and Alzheimer’s diseases, psoriasis or cancer [189]. Many of these compounds are long chain fatty acids that can be either saturated or unsaturated, polyunsaturated fatty acids (PUFAs) being the most studied for their pharmacological potential. These fatty acids have been found to have many benefits on human health, including protection against CRC [190,191,192]. Fish oils are the major source of n-3 PUFAs due to their high levels of EPA, docosahexaenic acid (DHA) and docosapentaenoic acid (DPA); however, their clinical use is often limited by their unpleasant fishy taste and their adverse effects [193] as well as their frequent contamination with heavy metals or the decline of global fish stocks [35]. Therefore, in recent years, microalgae have become a good alternative source of PUFAs such as EPA, DHA and DPA. Although these organisms have an elevated content in these compounds, bioengineering could produce huge amounts for pharmaceutical use. The most representative species of microalgae rich in fatty acids are *Tetraselmis* sp*.* and *Nannochloropsis oculata* [50,51,52,53,54].

A multitude of publications have revealed the therapeutic role of fatty acids in different types of inflammatory pathologies, such as Alzheimer’s disease [194], rheumatoid arthritis, lupus [195] or IBD [196]. An *in vitro* study on the colon adenocarcinoma cell line Caco-2 expressing GPR120 (G-protein coupled receptor with anti-inflammatory signaling properties after binding *n*-3 PUFAs) evidenced that EPA and DHA inhibited NF-κB activity and IL-1β secretion by activating ERK1/2 MAP kinase [50,197]. In the same line, DHA and DPAn-6 algal oils have been demonstrated to reduce TNF-α and IL-1β secretion as well as downregulate COX-2 expression and the subsequent PGE2 production in a human peripheral mononuclear cell line. These algal oils also reduced paw edema to an extent similar to indomethacin in rats, suggesting their anti-inflammatory properties [52]. *In vivo* studies have shown that a diet rich in EPA and DHA, found principally in oily fish, has potent anti-angiogenic effects mainly in colon, breast and prostate cancers, inhibiting production of many important angiogenic mediators such as vascular endothelial growth factor (VEGF), platelet-derived growth factor (PDGF), platelet-derived endothelial cell growth factor (PDECGF), COX-2, PGE2, NO, NF-κB, MMPs and β-catenin [54]. In this respect, van Beelen *et al.* [198] demonstrated the chemopreventive effects of an *n*-3 PUFA-rich microalgal oil diet in a similar extent that a fish oil-rich diet on AOM-induced colonic aberrant crypt foci (ACF) in mice, in comparison with corn oil diet. Thus, microalgal oil proved to be a good alternative to fish oil regarding protection against CRC. As mentioned above, EPA and DHA are *n*-3 fatty acids found mainly in fish oil [199,200] but also in microalgae [51,53]. Given EPA and DHA are not synthetized by animal tissues, they must be ingested with the diet. These fatty acids have been found to decrease vascular cell adhesion molecule 1 (VCAM-1), toll-like receptor 4 (TLR4), COX-2 and vascular endothelial growth factor receptor 2 (VEGFR2) expression as well as the production of other inflammatory mediators such as IL-6, IL-8, granulocyte macrophage colony-stimulating factor (GM-CSF), PGE2 and leukotriene B4 (LTB4) in IL-1β-induced human intestinal microvascular endothelial cells (HIMEC). Similarly, dietary intervention with fish oil rich in EPA and DHA significantly decreased colon production of PGE2 and LTB4, endothelial VCAM-1 and VEGFR2 in rats with colitis [201].

The first anti-inflammatory action of marine *n*-3 PUFAs found in humans was a reduction in generation of arachidonic acid (AA)-derived eicosanoids like PGE2 and LTB4 [202]. This effect correlated with a decrease in AA content, detected after a long period of intake of marine *n*-3 PUFAs [203]. EPA acts as an inhibitor of AA metabolism through COX and lipoxygenase (LOX) signaling pathways. In addition, EPA is a substrate for COX and LOX enzymes.

**Figure 2:**
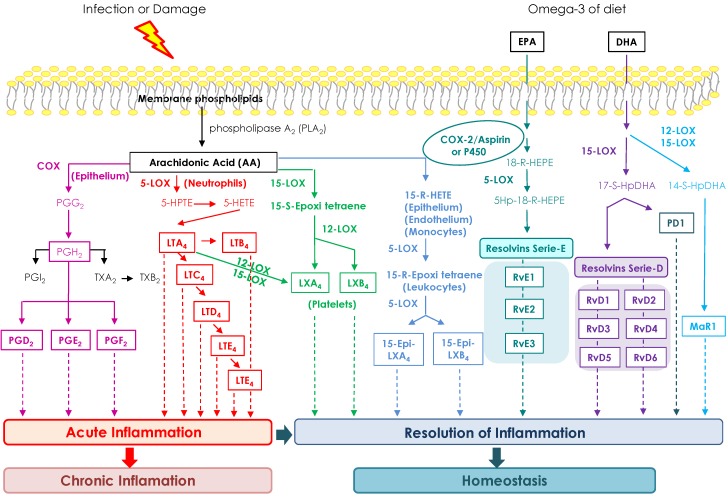
Resolution of inflammatory process after an infection or tissue damage. Biosynthetic pathways and actions of lipid mediators derived from arachidonic acid (AA), eicosapentaenoic acid (EPA) and docosahexaenoic acid (DHA).

In animal tissues, other families of lipid mediators derived from EPA and DHA are resolvins, protectins, lipoxins and maresins. Generically, all of them are oxidative PUFAs called oxylipins (OXLs). These OXLs are synthetized via COX and LOX and they are found to have anti-inflammatory and inflammation pro-resolving effects [204]. The resolution of inflammation is the key to restore tissue homeostasis and limit damage. In fact, Serhan *et al.* [205] demonstrated, for the first time, that this group of animal OXLs are synthetized during the inflammatory process and are able to promote the resolution of acute phase of inflammation. The main OXLs synthesis pathways in animals are shown in Figure 2. In microalgae, *n*-3 fatty acids-derived OXLs are produced from linoleic and α-linolenic acids and they have also shown a potent anti-inflammatory activity through decrease of inflammatory cytokines levels such as TNF-α [206] or amelioration of 2,4,6-trinitrobenzenesulfonic acid (TNBS)-induced colitis in rats [207]. In summary, the consumption of *n*-3 fatty acids such as EPA or DHA could have a beneficial effect in the resolution of inflammatory processes and thus prevent their progression to cancer. 

#### 3.2.3. Glycolipids

This type of complex lipids is widespread in nature especially in plants and bacteria, where they are mainly located in the thylakoid membranes of chloroplasts and cyanobacteria. Their general structure consists of a carbohydrate moiety that is beta-monogalactosyldiacylglycerol (MGDG) and digalactosyldiacylglycerol (DGDG) or alpha-linked sulfo-quinovosyl-acyl-glycerol (SQAG) to the sn-3 position of glycerol, which is acylated at the residual hydroxyls by fatty acids of different lengths and degrees of unsaturation [208]. They are present on the outer surface of eukaryotic cells membranes for cellular recognition as well as for providing energy or stability to the membrane [209]. The first evidence of the presence of glycolipids in microalgae was described in Gymnodinium, a genus of dinoflagellates (Dinophyceae class). The content of glycolipids in *Gymnodinium mikimotoi* was 17% of MGDG and DGDG, while in *Gymnodinium* sp. the proportion was double. Both compounds were shown to have hemolytic actions [210]. Several papers have revealed the high content of glycolipids in microalgae. In this regard, a study with the microalga *Pavlova lutheri* revealed that EPA was especially concentrated in MGDG (45%) as well as that DHA was dispersed within triacylglycerol (TAG) (27%), diacilphosphoglycerol (DPG) (22%) and betaine lipids (21%) [211]. Microalgae are rich in other chemical features of TAG. Xu *et al.* [212] performed a global characterization of different glycolipids from a marine diatom *Stephanodiscus* sp. In this study, 16 MGDGs, nine DGDGs, 23 SQDGs and eight phosphatidylglycerols (PGs) were identified. All of these lipids have shown a high potential as an alternative fuel source [213,214], but they could have an important role in pathologies related with NO signaling pathways, such as inflammatory diseases. In this line, methanolic extracts of *Tetraselmis chui* showed NO inhibitory activity on LPS-induced NO production in RAW264.7 macrophage cells [215]. A similar extract of the marine macroalga *Palmaria palmata* inhibited LPS-induced NO production in the same cell line through down-regulation of iNOS [216]. 

It is noteworthy that glycolipids from plants have shown anticancer activities in CRC. A glycolipid fraction from spinach containing mainly MGMG, MGDG, DGDG and SQDG inhibited DNA polymerase activity [217] and cell growth in human gastric cancer cells, as well as suppressed tumor growth in a tumor graft study [55]. In this line, the same glycolipid fraction caused a decrease in a solid adenocarcinoma (colon-26) tumor growth in mice without any side effects (loss of body weight or organ failure) by inhibition of angiogenesis and the expression of cell proliferation marker proteins such as Ki-67, PCNA and cyclin E in the tumor tissue [58,59]. Moreover, liposomes carrying surface-bound sialyl Lewis X (SLX) and containing MGDG (SLX-Lipo-MGDG) in nude mice bearing HT-29 human colon adenocarcinoma tumors showed a stronger and more promising suppression of solid tumor than MGDG alone [56]. In the same line, DGDG and SQDG from the Brown alga Sargassum *horneri* caused an apoptosis induction by fragmentation of DNA in Caco-2 colon cells [57]. All these studies of different glycolipids from plants or macroalgae demonstrate the potential of glycolipids as useful tools against CRC development.

More recently, an *in vivo* study in a young man demonstrated that the oils extracted from the microalgae *Nannochloropsis oculata* (rich in EPA conjugated to phospholipids and glycolipids but no DHA) might improve the bioavailable of EPA and DHA in human plasma in comparison with the oil from krill (rich in EPA and DHA). Moreover, this difference may be related to the different chemical constituents, especially the presence of glycolipids [218]. Glycolipids have been demonstrated to have bioactive effects in inflammatory processes involved with NO, but more studies are necessary to investigate this important role in inflammatory pathologies.

#### 3.2.4. Polysaccharides

Algae (micro- and macroalgae) produce polysaccharides with applications in the pharmaceutical and biomedical industries due to their biological activities: anti-inflamatory, antioxidant, antiviral, anticoagulant, anticancer and inmunomodulating [219]. However, the use of polysaccharides is restricted for various reasons, including a lack of simple methods for isolating them from extracts. Despite the fact that several methods for isolation of seaweed polysaccharides have been reported, all of them are rather work demanding and time consuming. In this respect, several microalgae secrete polysaccharides into the culture medium and these polymers are easily extracted [220].

Microalgae are excellent sources of polysaccharides whose beneficial activities have been demonstrated in various kinds of cell lines and animal models. These include polysaccharides from diatoms, chlorophytes, prasinophytes, haptophytes, rhodophytes, dinoflagellates and cyanobacteria [219]. There is a big amount of reports covering the antioxidant, anti-inflammatory, anticancer and immunomodulatory activities of polysaccharides derived from microalgae; nevertheless, there is little information on the effect of these compounds on IBD and CRC. The main results are summarized below. 

Microalgae polysaccharides are potent antioxidant. For example, polysaccharides extracted from *Isocrysis galbana* showed scavenging activity against superoxide and hydroxyl radicals [221]. Similarly, polysaccharides from *Pavlova viridis* and *Sarcinochrysis marina* Geitler and their degradation fragments showed antioxidant activity using 1,1-diphenyl-2-picrylhydrazyl (DPPH), hydroxyl-radical (OH) scavenging, lipid peroxidation inhibition and the mouse red blood cells (RBCs) hemolysis assay [222]. 

Pectic polysaccharides extracted from the plant *Rauwolfia verticillata* (Lour.) Baill.var.hainanensis Tsiang showed a potent anti-inflammatory effect on an experimental murine colitis model induced by DSS, decreasing the level of proinflammatory molecules such as activated NF-κB, TNF-α and IL-17 [223]. In the same line, dietary non-digestible polysaccharides had similar effects in the same experimental DSS mice model [224]. The effect could be related to restoring the balance between intestinal microbiota and mucosal immune responses. Their verified antioxidant and anti-inflammatory activities make these compounds very promising tools in IBD.

Microalgae polysaccharides have shown anticancer activity in several models, including CRC. An extracellular polysaccharide, GA3P (d-galactan sulfate, associated with l-(+)-lactic acid), synthesized by the toxic marine dinoflagellate *Gymnodinium* sp. A3, proved to be a potent inhibitor of DNA topo I and topo II irrespective of the presence or absence of the lactate group [225]. It inhibited growth of different cancer cell lines, as well as several colon cancer cell lines (HCC2998, KM-12, HT-29, WiDr, HCT-15 and HCT-116).

On the other hand, polysaccharides extracted from the microalgae *Aphanizomenon flos-aquae*, *Chlorella pyrenoidosa* and *Spirulina platensis* showed immunoestimulatory activity in THP-1 human monocytic cells [226]. The activity corresponded to high molecular weight polysaccharides that were named Immulina (for *Spirulina platensis*), Immunon (for *Aphanizomenon flos-aquae*) and Immurella (for *Chlorella pyrenoidosa*). Mice that consumed an extract containing Immulina displayed enhanced innate immune activity [227]. This immunoestimulatory effect has also been shown in other animal species and in humans [228]. These findings suggest the potential of these compounds as agents for immunotherapy in the treatment of cancer. 

#### 3.2.5. Proteins and Peptides

Due to their high protein content, microalgae are considered potential cell factories for both alimentary protein sources and for the production of therapeutic peptides and proteins [229]. Proteins from marine sources have many important and exclusive properties such as their film and foaming capacity, gel forming ability and antimicrobial activity [230]. Several therapeutic bioactivities have been described for phycobiliproteins from cyanobacteria and red algae, such as *Spirulina platensis* and *Porphyridium* sp.: hepatoprotective, anti-inflammatory, immunomodulating, antioxidant and anticancer effects [67,68]. For example, the phycobiliprotein C-phycocyanin isolated from *Spirulina platensis* caused the release of cytrochome C from mitochondria and caspase-dependent induction of apoptosis in HeLa cells [231]. In addition, C-phycocyanin mediated mitochondrial-dependent apoptosis in the DMH-induced rat model of colon carcinogenesis [232].

Peptides from microalgae also have interesting biological activities, especially anti-inflammatory [233] and anticancer activities [68,234]. A polypeptide from *Chlorella pyrenoidosa* has shown antitumor activity [69], and antihypertensive peptides have been isolated from *Chlorella vulgaris* and *Spirulina platensis* [235]. Cyanobacterial peptides with antitumoral activity include Symplocamide A from *Symploca* sp. [236], Somocystinamide A from *Lyngbya majuscule* [237], Apratoxin D from *Lyngbya majuscula* and *Lyngbya sordida* [238], Dragonamides C and D from *Lyngbya polychroa* [239] and Mitsoamide from *Geitlerinema* sp. [240].

With respect to microalgae-derived peptides, there are several studies in models related to CRC. *Nostoc* sp. produces the depsipeptides cryptophycins [241]. A synthetic analog of cryptophycin showed antiproliferative activity against HCT-116 human colon adenocarcinoma cells, causing inhibition of both DNA and RNA synthesis and subsequent G_2_/M arrest [242]. Desmethoxymajusculamide C (DMMC), a cyclic depsipeptide from the cyanobacterium *Lyngbya majuscula* exhibited antitumor activity against the human colon carcinoma cell line HCT-116 [243]; this effect was related to the disruption of microfilament networks. In addition, the same study showed that DMMC demonstrated efficacy on HCT-116 bearing SCID mice treated with 0.62 mg/kg daily for 5 days. Symplostatin isolated from the marine cyanobacterium *Symploca hydnoides* had antitumor effects in a murine solid tumor model, murine colon adenocarcinoma 38, maintained and tested in C57B1/6 mice [244]. Two peptidic proteasome inhibitors, carmaphycin A and B, exhibited strong cytotoxicity against lung and colon cancer cell lines [245]. In the same line, cyclic decapeptides isolated from *Anabaena minutissima* (UTEX 1613) exhibited antiproliferative activity against the HT-29 human colon cancer cell line [246]. Pitipeptolides, cyclic depsipeptides isolated from the marine cyanobacterium *Lyngbya majuscule* [247] and Grassypeptolide from *Lyngbya confervoides* [248], displayed cytotoxic activity against HT-29 cells, causing cell cycle arrest and inducing apoptosis. Pitipeptolides, Symplostatin, and other peptides that have cytotoxic activity against colon cancer cell lines, are dolastatin analogs (see below). 

Dolastatins are microtubule-disrupting agents produced by *Lyngbya* sp. and *Symploca* sp. [249]. They showed cytotoxic activity on a panel of human ovarian and colon carcinoma cell lines that was more potent than paclitaxel or vinblastine [250]. TZT-1027, a dolastatin 10 derivative, had antitumor activity against three murine solid tumors; colon 26 adenocarcinoma, B16 melanoma and M5076 sarcoma [251] and in human tumors xenografted in nude mice from colon (H-110, H-143) [252]. Dolastatin 10 was not successful as a single agent in phase II clinical trials, and new more potent analogs were produced, such as auristatin PYE [253]. This compound was less effective *in vitro* than dolastatin 10, but it had higher efficacy in two human colon adenocarcinoma models, DLD-1 and COLO 205. In addition, auristatin PYE potentiated the activity of cisplatin in a human colon tumor xenograft model [254]. A dolastatin obtained not from microalgae but from a marine mollusk (Dolastatin 15), along with the COX-2 inhibitor celecoxib, could prevent preneoplastic colonic lesions in a DMH-induced rat colon carcinogenesis model, through inhibition of NF-κB and iNOS [70], and it had anti-neoplastic effects in the same rat model through regulation of the phosphoinositide 3 kinase/Akt (PI3K/Akt) pathway [255]. Finally, two peptides produced by *Symploca* sp., largazole (histone deacetylase inhibitor) and dolastatin 10, showed cooperative activity against HT-29 colon cancer cells [256].

#### 3.2.6. Other Compounds

Cyanobacteria have been shown to be good producers of bioactive secondary metabolites with anticancer activity [257]. Some of them have shown activity against several colon cancer cell lines. The calothrixins are quinone-based natural products isolated from *Calothrix* cyanobacteria that show potent antiproliferative properties against several cancer cell lines [258]. Calothrixin B displayed antiproliferative activity against HCT-116 colon cancer cell line (IC_50_ 0.32 µM) [72]. Malyngamides are small amides produced by marine cyanobacteria; malyngamides isolated from *Lyngbya majuscule* (malyngamide C and 8-epi-malyngamide C) were found to be cytotoxic to HT29 colon cancer cells (IC_50_ 5.2 μM and 15.4 μM, respectively) [71]. Merocyclophanes A and B, isolated from a terrestrial *Nostoc* sp. (UIC 10022A), displayed antiproliferative activity against HT-29 cells (IC_50_ values of 3.3 and 1.7 µM, respectively) [259]. Hierridin B, a secondary metabolite (polyketide) isolated from the marine cyanobacterium *Cyanobium* sp. LEGE 06113, tested in a panel of eight human cancer cell lines, showed selective cytotoxicity towards HT-29 cells, although with moderate potency (IC_50_ 0.1 mM) [260].

Other microalgae-derived compounds with therapeutical potential in IBD and CRC, due to their antioxidant activity, include tocopherols from *Porphydium* sp. and *Spirulina platensis* [70], and phenolic compounds (benzoic acid and cinnamic acid derivatives, hydroxybenzldehydes) from *Spirulina maxima*, *Chlorella ellipsoidea* and *Nannochloropsis* sp. [73,74].

## 4. Inflammation and Skin Cancer

### 4.1. Molecular Pathways of Skin Carcinogenesis

Skin is considered the largest organ in the body and acts as a physiological barrier that protects us from the deleterious effects of solar UV radiation, which is the main cause for skin cancer. This cancer is currently the most common type of human cancer worldwide [261]. Of the UV light, which reaches the Earth, 5% is UVB while the remaining 95% is UVA. UVB only penetrates into the epidermis and can induce a variety of both acute and chronic adverse effects, including skin photoageing, inflammation, immunosuppression and direct DNA damage with signature mutations predisposing to skin cancers [262]. UVA can penetrate deeper into the underlying dermis and, in comparison to UVB, is less damaging, being related to ageing skin events. Moreover, it can damage DNA indirectly through the generation of free radical and oxidative injury [263].

Exposure to UV radiation is associated with the development of approximately 65% of melanoma cases and nearly 90% of non-melanoma skin cancers (NMSC) [264]. NMSC is the most common form of cancer diagnosed in the white-skinned population. Its incidence has increased dramatically worldwide, developing even in younger age groups. The two major types of NMSC comprise basal cell carcinoma (BCC) and squamous cell carcinoma (SCC), and account for approximately 80% and 20% of all skin cancers, respectively. Almost all cases of NMSC arise on sun-exposed areas of skin, like the face, ears, neck and back of the hands, implicating chronic exposure to solar UV radiation as the major etiologic factor [265]. BCC is the most common easily treated form of skin cancer in Europe and the United States with an estimated average annual incidence of 100 per 100,000 inhabitants [266]. The risk of BCC seems to be similar for young males and females, but after the age of 45 years, men are twice more likely to present BCC than women. BCC tumors grow slowly (months to years), are locally invasive and rarely metastasize (0.003%–0.55%) or cause death [267].

SCC, the second most common type of skin cancer, is a tumor with local destructive properties and men have a three times higher risk than women at all ages. This cancer mainly occurs in the centro-facial region in people with fair skin, blond or red hair and blue or green eyes. In contrast to BCC, SCC tends to grow more rapidly (weeks to months) and if untreated, it can become locally invasive, leading to metastases in about 5% of all cases [268]. The highest risk factor is the presence of actinic keratosis (AK), which can progress to SCC in 5%–10% of cases. [269]. AKs develop in sun-exposed areas, including the face, forehead, scalp, neck and dorsum of the hands. AKs are small lesions (3–6 mm) characterized by the formation of rough or scaly macules, papules, or plaques of reddish/brown-color on an erythematous base [270]. They are characterized by dysplastic keratinocyte lesions confined to the basal epidermal layer of the skin. If untreated, up to 10% of AKs can undergo malignant transformation and progress to SCC. In rarer instances, AKs may also turn into BCC [271]. On the other hand, AKs have also been shown to regress spontaneously [272]. Currently, authorities debate whether AK should be considered as SCC *in situ* or as a premalignant precursor to invasive SCC. However, it is clear that they represent an intermediate stage in a multistep evolution of SCC. The risk of transformation of AK to SCC (invasive or *in situ*) increases with rapid enlargement, bleeding, erythema, ulceration, pain, inflammation and diameter greater than 1 cm, as well as in immunocompromised patients [273]. Since it is not possible to predict whether a given AK will regress, persist, or develop into SCC, early treatment of AK is recommended. The current interventions available can be broadly grouped into two categories: individual lesion-based treatments, including curettage and cryotherapy with liquid nitrogen, and field-directed therapies, such as photodynamic therapy and topical therapies (5-fluorouracil, diclofenac, imiquimod or ingenol mebutate), targeted at multiple lesions over a large area. However, topical therapies have long treatment durations, typically up to 16 weeks, and can elicit local skin responses, as erythema, ulcerations, and crusting [274].

Skin inflammation can be caused by mechanical injuries, chemical or biological agents, immunologic disorders and physical agents, such as UV radiation. Epidemiological studies and numerous animal models, such as those of UV-induced or chemically induced skin carcinogenesis, provide strong evidence for a critical link between the inflammatory microenvironment and the development and progression of cancer [275]. The connection between inflammation and skin cancer can be viewed as consisting of two pathways: the intrinsic and extrinsic pathways. In the intrinsic pathway, genetic mutations by UV-induced DNA damage can lead to inactivation of tumor suppressor genes, such as p53, p16 INK4a and p14ARF [276] or activation of oncogenes, including RAS oncogenes (HRAS, KRAS and NRAS) and BRAF [277]. In the extrinsic pathway, exposure of skin to chemical, physical or biological agents induces an inflammatory response that includes vasodilation, microvascular structural changes and escape of plasma proteins from the bloodstream, and transendothelial migration of leukocytes at the site of tissue injury [278]. Neutrophilic infiltration induces ROS and RNS production, which can directly damage chromatin and promote DNA mutagenesis or initiate intercellular transduction pathways leading to inflammation and tumor formation [279]. 

The two pathways converge, resulting in the stimulation of intracellular signal transduction pathways, including mitogen-activated protein kinases (MAPK), PI3K/Akt and Janus kinase/signal transducers and activators of transcription (JAK/STAT). Subsequently, several transcription factors are activated, such as NF-κB, STAT3 and hypoxia-inducible factor-1 alpha (HIF-1α). These transcription factors coordinate the production of numerous inflammatory mediators, such as cytokines, chemokines and surface adhesion molecules, as well as the synthesis of COX-2 or iNOS [280]. Excessive production of proinflammatory factors leads to chronic inflammation, which in turn may increase the risk of tumorigenesis. Therefore, pharmacological control of the inflammatory response of the skin to solar radiation may be beneficial in decreasing the incidence of skin cancer.

MAPKs regulate the actions of a variety of downstream transcription factors involved in cancer development [281,282]. Previous papers have demonstrated a critical role for the MAPK pathway in UVB-induced skin damage and skin cancer. In the human keratinocyte HaCaT cell line, UVB exposure induced the phosphorylation of ERK, JNK and p38 MAPKs, with the main signaling pathways being that activated by UV radiation in these cells [283]. Similarly, *in vivo* studies have shown an enhanced phosphorylation of Erk1/2, p38 and JNK in UVB-induced inflammation in SKH-1 hairless mice skin [284] as well as in the 9,10-dimethylbenz[a]anthracene (DMBA)/12-*O*-tetradecanoylphorbol-13-acetate (TPA)-induced skin inflammation and carcinogenesis model [285]. PI3K induces the production of phosphatidylinositol (3,4,5)-trisphosphate, which is responsible for the translocation of Akt to the plasma membrane [286]. Activated Akt regulates multiple biological processes such as cell survival, proliferation and growth. It has been reported that dysregulation of the PI3K/Akt signaling pathway is involved in a number of human diseases, including cancer [287]. In this line, the upregulation of PI3K expression and Akt phosphorylation has been demonstrated in different animal models of skin tumorigenesis, including UVB-exposed SKH-1 hairless mice [288] and chemically induced skin cancer [289]. The JAK/STAT pathway is a signaling pathway employed by a large number of cytokines, growth factors and hormones upon binding their specific receptors. Receptor-mediated tyrosine phosphorylation of Jak family members activates STAT proteins, which in turn directly bind DNA and regulate gene transcription [290]. These proteins and particularly STAT3 are highly increased in most malignancies and regulate the expression of a wide range of genes that contribute to skin carcinogenesis [291]. 

As regards to inflammasomes, it has been shown that one of the earliest responses of keratinocytes against UV damage is the formation of the inflammasome complex within the cytoplasm. In HaCaT keratinocytes, UVB radiation induces rapid ATP release from dying cells into the extracellular space [292], leading to activation of NLRP3 and the subsequent increase in IL-1β and IL-18 levels [92]. Prolonged IL-1β production after UV-exposure results in tissue damage that can lead to a microenvironment of chronic inflammation that promotes cancer development [293]. However, IL-18 represents a double-edged sword in cancer, since its activation may stimulate tumor development [294] or oppositely, increase anti-tumor immunity and limit tumor growth through the activation of natural killer cell responses [295]. The influence of inflammasomes in the carcinoma development has been shown to have contrasting roles. A previous study has demonstrated that IL-1 receptor- or caspase-1-deficient mice present a reduction in tumor incidence and number in comparison to wild type animals in a model of chemically induced skin carcinogenesis. These authors also reported that mice specifically deficient in ASC in myeloid cells show a reduced tumor incidence; however, ASC-deficient mice in keratinocytes developed more tumors than controls in the same model of skin cancer [296]. Another recent paper using ASC or caspase-1 knockout mice supports the function of these proteins in the protection against SCC tumorigenesis and progression [297]. 

In skin, autophagy is believed to be a form of an endogenous defense mechanism against mechanical stresses, chemical or biological agents and UV radiation. Consequently, this process is closely related to skin homeostasis and may have a critical role in the development and progression of skin diseases [298]. A recent study supporting the tumor-suppressive function for autophagy has reported that the activation of this process by serum starvation in SCC cells reduced cell growth and senescence [299]. However, other investigations reported the protumoral activity of autophagy in skin cancer. In this line, an increased expression of the autophagy marker LC3 has been demonstrated in patients with SCC, which was associated with disease progression and prognosis of cancer [300]. Interestingly, an *in vitro* study showed that the inhibition of both Akt signaling and autophagy by the lysosomal inhibitor chloroquine enhanced the susceptibility of metastatic SCC cells to docetaxel-induced apoptosis [301]. 

Sirtuins have an important function in cellular pathways associated with skin structure and function, including UV-induced premature skin ageing, inflammation and cancer [302]. In this line, SIRT1 expression was decreased in human skin keratinocytes after exposure to UV radiation or H_2_O_2_. Interestingly, treatment with resveratrol, a SIRT1 activator, protected against UV or H_2_O_2_-induced apoptosis, suggesting that these activators could be used as new skin anti-aging strategies [303]. Accumulating evidence indicates that the role of SIRT1 in skin cancer is complex. Recently, a dual role of SIRT1 in a model of UVB-induced skin cancer was reported. In this paper, keratinocyte-specific heterozygous deletion of SIRT1 enhanced tumorigenesis, whereas homozygous deletion of this gene inhibited tumor development [304]. These authors also reported a decrease in SIRT2 protein levels in human SCC from sun-exposed areas. In addition, this study confirmed the tumor suppressor effect of this sirtuin since SIRT2 knockout mice presented an increased tumor growth in DMBA/TPA-induced skin carcinogenesis [305]. However, overexpression of SIRT1 was observed in patients with AK or SCC, with no difference between both pathologies, suggesting that this protein may have a function in the early stages of skin carcinogenesis [306]. Similarly, a more recent study reported an upregulation of gene expression of the seven SIRT family members in epidermal human cells, including HaCaT keratinocytes and A431 epidermoid carcinoma cells as well as in human AK or SCC skin biopsies [307]. 

### 4.2. Microalgae as a Source of Biomolecules with Potential in Inflammation and Skin Cancer

#### 4.2.1. Carotenoids

##### 4.2.1.1. β-Carotene

Carotenoids are important compounds with anti-oxidant potential whose structure and physicochemical properties could be implicated in their skin protective effects against sunlight damage [308]. Previous *in vitro* studies, investigating the antioxidant potential of β-carotene, have shown that this carotenoid reduced the photoinactivation of the enzymes catalase and superoxide dismutase as well as protein cross-linking. β-carotene has also been reported to decrease UVA-induced heme oxigenase 1 (HO-1) expression in skin fibroblasts [309]. A recent paper evidenced that human dermal fibroblasts incubated with an extract enriched in β-carotene and stressed with H_2_O_2_ showed an increase in cell viability and a reduction of intracellular amount of ROS, membrane lipid peroxidation and DNA damage. Moreover, this compound improved mitochondrial functionality, enhancing the basal respiration of mitochondria, and promoted the regenerative capacity of cells after exposure to pro-oxidant stimuli [310]. 

The anti-inflammatory and photoprotective activity of β-carotene has also been reported in different *in vivo* models. The effects of this compound were evaluated in a mouse ear edema model induced by capsaicin or mustard oil, which promoted non-neurogenic edema formation. This study showed that the edema, swelling and myeloperoxidase (MPO) activity were gradually decreased after administration of β-carotene [311]. 

Different papers have reported that supplementation with β-carotene protects the skin against UV-induced erythema in humans. Along these lines, increasing doses of β-carotene for 24 weeks significantly reduced serum lipid peroxidation after exposure to UV radiation [312]. Another study was conducted in healthy females over the age of 50 years to evaluate the effects of dietary β-carotene at two different doses for 90 days on wrinkles, elasticity and UV-induced DNA damage. The results showed that only the lowest dose of this carotenoid was able to prevent and repair photoaging [313]. As regards skin cancer, a case-control study in NMSC patients reported that the incidence of cancer in these patients was inversely related to the level of serum β-carotene and dietary ingestion of this carotenoid [314]. 

##### 4.2.1.2. Astaxanthin

The xanthophyll carotenoid astaxanthin has been reported to have a higher preventive effect toward photo-oxidative changes than other carotenoids, such as β-carotene or α-tocoferol. Camera *et al.* [315] reported the photoprotective role of astaxanthin on UVA-related damage in human dermal fibroblasts, through a reduction in ROS levels and HO-1 expression as well as an increase in antioxidant enzyme activities. The skin anti-aging effects of this carotenoid have also been related to the downregulation of MMP-1 and skin fibroblast elastase [316]. In addition, astaxanthin decreased the UVB- and UVC-induced inflammatory mediator expression, including iNOS, COX-2, macrophage migration inhibitory factor, IL-1β and TNF-α in HaCaT keratinocytes as well as inhibited apoptosis by reducing Bax, Bcl-2 and caspase-9 [317].

An *in vivo* experiment showed that liposomes containing astaxanthin, topically applied to the dorsal skin of hairless mice, prevented UV-induced stratum corneum thickening and inhibited collagen-degrading enzymes, thus reducing wrinkle formation. Moreover, this liposomal formulation avoided melanin production in the skin after UV exposure [318]. Astaxanthin has also been demonstrated to have anticancer activity *in vivo*. This compound delayed the formation of papillomas and reduced the average number of papillomas per mouse in DMBA/TPA-induced skin carcinogenesis model [319]. In the same line, the effects of astaxanthin and astaxanthin esters from *Haematococcus pluvialis* on UV-DMBA-induced skin cancer in rats have been reported. Interestingly, results from this study observed that astaxanthin esters were more potent than astaxanthin in reducing tumor incidence and growth, by more strongly inhibiting the accumulation of ROS and tyrosinase enzyme activities. The better antitumoral activity of esters could be due to their increased bioavailability [320].

Cosmetic effects of astaxanthin have been studied in humans. An open-label non-controlled test conducted in healthy females evidenced that oral supplementation of astaxanthin isolated from *Haematococcus pluvialis* combined with its topical application improved skin wrinkle, elasticity and moisture content of corneocyte layer after 8 weeks of treatment. Similarly, a randomized double-blind placebo controlled study with healthy males demonstrated that the supplementation with this carotenoid for 6 weeks also improved the skin condition in males [321].

##### 4.2.1.3. Lutein

The photoprotective effects of lutein, a xanthophyll carotenoid naturally present in microalgae, have been previously studied *in vitro*. Pre-treatment with lutein protected a human keratinocyte cell line (CDD 1102 KERTr) and primary human keratinocytes from foreskins against UVB-induced cell damage through increased cell viability and proliferation, and reduced apoptosis [322].

Lutein has shown anti-inflammatory effects in a mouse ear edema model induced by capsaicin or mustard oil via a marked reduction in swelling and neutrophil infiltration [311]. In relation to cancer, this molecule exhibited antitumor-promoting activity on two-stage mouse skin carcinogenesis model, through inhibition of peroxynitrite-induced damage [319]. In addition, a photocarcinogenesis model conducted in SKH-1 hairless mice fed lutein/zeaxanthin-supplemented diet showed a decreased tumor multiplicity and total tumor volume, in comparison with UVB-irradiated control animals fed the standard diet [323].

In humans, supplementation with capsules rich in lutein and other carotenoids such as β-carotene provided moderate protection against erythema induced by UVB in healthy adult volunteers [324].

##### 4.2.1.4. Zeaxanthin

A previous *in vitro* study supporting the antitumor potential of zeaxanthin reported that this compound inhibited PDGF-induced skin fibroblast migration and PDGFR-β and MAPKs phosphorylation in a concentration-dependent manner [325].

The acute photoprotective capacity of zeaxanthin has been demonstrated in hairless SKH-1 mice fed lutein plus zeaxanthin-enriched diet for 2 weeks prior to UVB exposure. Dietary carotenoids reduced the edematous cutaneous response and the percentage of PCNA and bromodeoxyuridine positive cells, demonstrating that the presence of these antioxidants in the skin may diminish the deleterious oxidant effect of irradiation [326].

A double-blind, placebo-controlled study in human subjects showed that the combined oral and topical administration of lutein and zeaxanthin supplied a higher antioxidant protection, measured by changes in lipid peroxidation and photoprotective activity, than administration of these carotenoids individually [327].

##### 4.2.1.5. Fucoxanthin 

The marine carotenoid fucoxanthin has shown important antioxidant and photoprotective activity in different *in vitro* models. Along these lines, fucoxanthin isolated from *Sargassum siliquastrum* reduced UVB-induced intracellular ROS production, cell damage and apoptosis in human fibroblast in a dose-dependent manner [328]. In a similar way, this compound attenuated the levels of ROS in H_2_O_2_-treated HaCaT keratinocytes as well as quenched the hydroxyl radical generated by the Fenton reaction in a cell-free system. Moreover, the protective effects of fucoxanthin against oxidative damage were related to inhibition of apoptosis through down-regulation of Bax expression and increase of Bcl-2 expression [329]. More recently, Zheng *et al.* [330] studied the antioxidant activity of fucoxanthin in HaCaT cells and showed that this treatment increased the expression of glutamate-cysteine ligase catalytic subunit (GCLC) and glutathione synthetase (GSS), two enzymes involved in reduced glutathione (GSH) synthesis. The mechanisms underlying these effects were related to activation of the transcription factor Nrf2 via PI3K/Akt, which in turn activated the transcription of ARE-driven GCLC and GSS genes, leading the synthesis of antioxidant GSH.

The anti-aging effects of fucoxanthin have also been evaluated in animal models. Topical pre-treatment with fucoxanthin from *Undaria pinnatifida* for 10 weeks tended to diminish the number of wrinkles in UVB-irradiated chronically dorsal skin of hairless mice. This pigment reduced UVB-induced epidermal hypertrophy as well as VEGF and MMP-13 expression in the epidermis, which are known to induce angiogenesis [331]. Another paper showed that topical and oral application of fucoxanthin to UVB-irradiated male hairless mice exhibited anti-melanogenic activity via suppression of PGs synthesis and expression of melanogenic receptors in melanocytes [332].

In conclusion, the use of carotenoids in the diet in combination with topical application may play an important role in skin photoprotection, thus being a useful strategy for treatment of skin diseases caused by excessive sun exposure.

#### 4.2.2. Fatty Acids

As mentioned above, it is well known that PUFAs play important roles in many physiological and pathological processes. In the last years, their protective effects on acute and chronic skin diseases have been described, which are related to modulation of membrane fluidity and immune function, contribution to barrier integrity, promotion of wound healing and inhibition of eicosanoids and cytokines production, among others [333,334,335,336,337]. 

Previous *in vitro* studies have shown the anti-inflammatory effects of n-3 PUFAs, like EPA or DHA, in skin cells through inhibition of the production of UVB- and TNF-α-induced IL-8 in HaCaT keratinocytes and CCD922SK fibroblast cell line [338]. Similarly, the treatment of human keratinocytes with n-3 PUFAs through liposomes based on a natural marine lipid extract reduced the croton oil-induced inflammatory response by downregulating PGE2 and IL-8 levels [339]. It has been suggested that the induction of COX-2 by EPA in HaCaT keratinocytes, via peroxisome proliferator-activated receptor gamma (PPARγ) activation, may have an important role in the anti-inflammatory and protective mechanism of action of PUFAs [340]. Conversely, Obata *et al.* [341] described that EPA reduced expression of COX-2 and the subsequent PGD2 generation in human mast cells. On the other hand, n-6 PUFAs were shown to increase PGE2 levels, which was related with keratinocytic skin cancer growth [342,343]. Another mechanism involved in the anti-inflammatory effect of EPA includes the inhibition of ERK and JNK in HaCaT cell line, resulting in decreased MMP-1 expression [344]. A recent study describes that EPA increases the expression of aquaporin-3, a water/glycerol transport protein, in HaCaT cells through ERK activation, leading to a reduction in UVB-induced photodamage [345].

The anti-inflammatory activity of PUFAs has also been reported in different *in vivo* skin models. In hairless mice, topical treatment with the *n*-3 PUFA 11,14,17-eicosatrienoic acid for 3 days reduced UV-induced epidermal and dermal thickness and inflammatory cell infiltration, as well as attenuated the expression of IL-1β, COX-2 and MMP-13 [346]. In a recent study, rats receiving oil mixes with *n*-9, *n*-6 and *n*-3 PUFAs and subjected to thermal burn showed a reduction of the thermal lesions and a decrease in NF-κB activity and cell proliferation [347]. As regards to cancer, the effects of high-fat diets rich in *n*-6 PUFAs or *n*-3 PUFAs were evaluated on UVB-induced skin carcinogenesis in hairless mice. The results reported an enhanced tumorigenesis in mice fed *n*-6 PUFAs; on the contrary, animals receiving *n*-3 PUFAs presented a reduced tumor incidence and multiplicity [348,349]. 

Different clinical studies have reported that supplementation with PUFAs protects the skin against UV-induced erythema. A double-blind, randomized study reported that the oral administration of EPA for 3 months reduced UVB-induced PGE2 levels in healthy skin [350]. More recently, it has been shown that EPA-rich *n*-3 PUFAs abrogated photoimmunosuppression in human skin, reduced sunburn sensitivity and prevented DNA damage, providing support for their chemopreventive role [351,352]. These authors conducted another double-blind, randomized controlled study to evaluate the mechanisms underlying the photoprotective effect of EPA. Supplementation with this PUFA for 12 weeks increased the ratio EPA/AA in the skin, shifting eicosanoid production towards less pro-inflammatory mediators [353].

A prospective cohort study evaluated associations between plasma phospholipid levels of *n*-3 and *n*-6 PUFAs and the development of NMSC. The results showed a reduced risk of SCC in patients with high EPA concentrations and omega-3/-6 ratio and an inverse association of total omega-6 and BCC risk [354]. However, a study of over 40,000 American male health professionals failed to find a link between *n*-3 PUFA intake and BCC risk [355]. In conclusion, the photoprotective potential of *n*-3 PUFAs against UV radiation-induced inflammation and potentially longer-term photodamage should be further explored.

#### 4.2.3. Glycolipids

Glycoglycerolipids such as MGDG, DGDG and SQDG can be widespread in microalgae, as previously mentioned. In general, they have demonstrated to have remarkable biological effects, including inhibition of *in vitro* and *in vivo* tumor-promoting-activity [356] as well as anti-inflammatory and immunosuppressive effects [357].

The effects of microalgae glycolipids against skin inflammation and cancer have been largely studied, and their mechanisms have been suggested by using *in vitro* cell cultures as well as *in vivo* animal models. A previous *in vitro* study demonstrated that pretreatment with glycoglycerolipid analogues was able to diminish phorbol 12-myristate 13-acetate (PMA)-promoted tumorigenicity in human dermal fibroblasts through protein kinase C (PKC)-dependent mechanisms [358].

In addition, these compounds have been demonstrated to have anti-inflammatory activity *in vivo*. The effects of different glycoglycerolipids from the thermophilic blue-green alga ETS-05 were evaluated in croton-oil-induced ear edema and carrageenan-induced paw edema models. Topical pre-treatment with MGDG, DGDG and SQDG inhibited the croton-oil-induced edema, with MGDG being the most potent anti-inflammatory glycolipid. Similarly, these findings were confirmed in the carrageenan-induced paw edema model [357].

As regards to skin cancer, Colombo *et al.* [359] evaluated the antitumoral activity of glycoglycerolipids analogues in an *in vivo* two-stage mouse skin carcinogenesis model, using DMBA as an initiator and TPA as a promoter. All compounds tested delayed the formation of papillomas and reduced the number of papillomas per mouse. More recently, these authors confirmed the antitumor-promoting effects of other synthetic glycoglycerolipids on mouse skin in the same model [208]. Currently, this kind of natural compounds could have a promising employment in the field of prevention or treatment of cancer.

#### 4.2.4. Polyssacharides

Revising literature, there is only one paper showing the anti-inflammatory properties of sulfated polysaccharides derived from red microalgae in *in vitro* and *in vivo* models of skin inflammation. The *in vitro* assay showed that these polysaccharides suppressed polymorphonuclear leukocyte migration and partially inhibited their adhesion to endothelial cells. In human volunteers, topical application of polysaccharide material inhibited cutaneous erythema induced by a known irritant [64].

#### 4.2.5. Proteins

As mentioned above, microalgae are considered an important source for protein production. Phycobiliproteins from *Spirullina platensis* have been shown to have important activities, including antioxidant, anti-inflammatory and neuroprotector [360,361] actions. In this regards, the phycobiliprotein C-phycocyanin isolated from *Spirulina platensis* prevented TPA-induced tumor promotion in mouse skin through reduction in COX-2, IL-6 and pSTAT3 expression [362]. 

Some photosynthetic marine organisms have evolved mechanisms to counteract UV-radiation by synthesizing UV-absorbing compounds, called mycosporine-like amino acids (MAAs). MAAs from marine organisms, with only two exceptions, are imine derivatives of mycosporines that contain an amino-cyclohexenimine ring linked to an amino acid, amino alcohol or amino group [363], and they are biosynthesized by shikimic acid pathway for the synthesis of aromatic amino acids [364]. In addition, MAAs have been identified in a number of taxonomically diverse organisms such as fungi, eukaryotic marine invertebrates and a wide variety of other marine organisms [365]. Around 20 members can be found in the MAA family, including mycosporine-glycine, palythine, palythinol, asterina-330, porphyra-334 and shinorine. In microalgae species, shinorine is the most frequent and abundant MAA [366]. MAAs have been reported to have a high photostability [367], acting as endogenous antioxidant that protect microalgae against UV-induced damage [368,369,370]. The anti-photoaging role of MAAs has been evaluated in different skin cell lines. In this regards, porphyra-334 from *Porphyra* (*P.*) *yezoensis* inhibited ROS production and MMPs expression in UVA irradiated human skin fibroblasts [371]. Recently, MAAs isolated from the marine green alga *Chlamydomonas hedleyi* have demonstrated to have anti-inflammatory activity in HaCat cells, by reducing COX-2 and involucrin expression [372].

#### 4.2.6. Other Compounds

Due to their antioxidant activity, phenolic and phenolic-derived compounds, which can be obtained from microalgae [73,74,75], could be used to control the inflammatory response of the skin to solar radiation and to prevent skin cancer. The structures of natural polyphenols vary from simple molecules (such as phenolic acids) and other simple polyphenolic compounds (such as flavonoids), to the more complex phlorotannins, which consist of polymeric structures made up of units of phloroglucinol (1,3,5-trihydroxybenzene), typically isolated from marine brown algae or Phaeophyceae [373]. Many of them have been reported to show anti-melanoma or anti-skin cancer activities [374]. For example, dietary feeding and topical application of brown algae polyphenols had a protective effect against UVB radiation-induced skin carcinogenesis in SKH-1 mice [375]. Polyphenol-containing aqueous methanol extract of the red alga *Porphyra yezoensis* was shown to modulate viability and apoptosis of the UVB-exposed HaCaT cells [376]. With respect to phlorotanins, their capacity to inhibit UV-induced oxidative stress and gelatinase activity makes them potential anti-photoaging agents [377]. 

Microalgae have been shown to be an important source of tocopherol, a fat-soluble phenolic compound with varying degrees of vitamin E antioxidant activity [378,379]. A previous *in vitro* study showed that tocopherol decreased cell proliferation and activation of MAPKs and NF-κB signaling induced by argemone oil in HaCaT cells [380]. γ-tocopherol has been shown to have the most important anti-inflammatory activity and it can reduce PGE2 levels *in vitro* [381] and *in vivo* [382,383]. A hydrophilic γ-tocopherol derivative, γ-tocopherol-*N*, *N*-dimethylglycinate hydrochloride, reduced photoinflammation in mouse skin, by decreasing COX-2 expression [384].

The anti-inflammatory and antitumoral effects of tocopherol have also been reported in different animal models. Topical administration of d-α-tocopherol acetate to the skin of hairless mice reduced erythema, edema and skin sensitivity associated with UVB-induced sunburn [385]. Moreover, vitamin E application 30 minutes prior to TPA treatment suppressed early inflammatory and oxidative stress responses induced by TPA through inhibition of H_2_O_2_, MPO activity and lipid peroxidation [386]. Regarding the tumor suppressive effects, topical application of vitamin E to mice for 3 weeks prior to UV irradiation reduced cancer formation and immunosuppression by UV radiation in hairless mice [387].

Microalgae could provide a rich source of ascorbic acid in our diet, despite the differences in the composition found in microalgae species (0.11%–1.62% ascorbic acid) [388]. To solve the problem of its high instability, liposoluble vitamin C derivatives, such as tetra-isopalmitoyl ascorbic acid, are often used in dermocosmetic products [389]. *In vitro* studies have reported important biological activities for vitamin C. For example, vitamin C and vitamin E have shown to have preventive effects against deleterious cutaneous cell damage caused by a herbicide in HaCaT keratinocytes [390]. In addition, vitamin C antagonized UV-induced apoptosis and activated tumor suppressor genes in human skin cancer cells [391]. Ascorbic acid and its derivatives have been shown to protect against sunburn in mice, delay the onset of skin tumors and reduce UV-induced skin wrinkling [392].

Another of the main active compounds in marine micro and macroalgae include the sterols, such as fucosterol [393], which showed antioxidant activity and cytotoxicity against several cancer cell lines [394]. Fucosterol protected human dermal fibroblast against UVB-induced damage, decreasing the expression of MMP-1, IL-6 and other inflammatory markers, and increasing the production of type I procollagen and TGF-β1 [395,396]. These results suggest the potential of fucosterol for preventing skin ageing and development of skin cancer. 

## 5. Conclusions

Numerous animal models and epidemiologic studies support the role of a deregulated inflammation in tumor development. In this regard, there are many triggers of chronic inflammation that increase the risk of cancer, such as immunological diseases (for example, IBD is associated with colon cancer) and cutaneous lesions associated with chronic exposure to UV radiation (for example, AK is related to skin cancer). There are two different pathways linking inflammation and cancer: an extrinsic pathway mediated by chronic inflammation and oxidative environment (inflammation-cancer) and an intrinsic pathway in which genetic mutations cause alterations in cell proliferation, reduction of apoptosis, or increase in metastasis and angiogenesis; these effects in the absence of an underlying inflammation, initiate a tumor-driven host immune response leading to a microenvironment composed of inflammatory cells (cancer-inflammation). These data confirm the role of inflammatory processes in cancer origin and development.

New strategies for the prevention and treatment of inflammatory diseases and associated colon and skin cancers should be an important focus in cancer research. A high number of *in vitro* and *in vivo* studies and some clinical data have established the antioxidant, anti-inflammatory and antitumor potentials of microalgae species and their products, including carotenoids, fatty acids, glycolipids, polysaccharides and proteins. In this line, the simple growth requirements of microalgae make them attractive to meet the demands of the pharmaceutical, food and cosmetic industry. Complementary basic studies and well-controlled randomized clinical trials are needed to establish the optimal dose for chemopreventive efficacy, excluding toxic effects, all with the objective to be validated for a good clinical practice.

## References

[1] Kansu E. (2014). ISH 2014 World Congress: Report of the Chair of Council. Hematology.

[2] Balkwill F.R., Mantovani A. (2012). Cancer-related inflammation: Common themes and therapeutic opportunities. Semin. Cancer Biol..

[3] Sue S., Shibata W., Maeda S. (2015). Helicobacter pylori-induced signaling pathways contribute to intestinal metaplasia and gastric carcinogenesis. Biomed. Res. Int..

[4] Wang G.Z., Cheng X., Li X.C., Liu Y.Q., Wang X.Q., Shi X., Wang Z.Y., Guo Y.Q., Wen Z.S., Huang Y.C. (2015). Tobacco smoke induces production of chemokine CCL20 to promote lung cancer. Cancer Lett..

[5] Prasad R., Katiyar S.K. (2014). Ultraviolet radiation-induced inflammation activates β-catenin signaling in mouse skin and skin tumors. Int. J. Oncol..

[6] Malhotra P., Anwar M., Nanda N., Kochhar R., Wig J.D., Vaiphei K., Mahmood S. (2013). Alterations in K-ras, APC and p53-multiple genetic pathway in colorectal cancer among Indians. Tumour Biol..

[7] Karahan B., Argon A., Yıldırım M., Vardar E. (2015). Relationship between MLH-1, MSH-2, PMS-2,MSH-6 expression and clinicopathological features in colorectal cancer. Int. J. Clin. Exp. Pathol..

[8] Coussens L.M., Werb Z. (2002). Inflammation and cancer. Nature.

[9] Motilva V., García-Mauriño S., Talero E., Illanes M. (2011). New paradigms in chronic intestinal inflammation and colon cancer: Role of melatonin. J. Pineal Res..

[10] Wang F., Arun P., Friedman J., Chen Z., van Waes C. (2009). Current and potential inflammation targeted therapies in head and neck cancer. Curr. Opin. Pharmacol..

[11] Sporn B. (2011). The Big C for chemoprevention. Nature.

[12] Gravitz L. (2011). First Line of defence. Nature.

[13] Demaria S., Pikarsky E., Karin M., Coussens L.M., Chen Y.C., El-Omar E.M., Trinchieri G., Dubinett S.M., Mao J.T., Szabo E. (2010). Cancer and inflammation: Promise for biologic therapy. J. Immunother..

[14] Baron J.A. (2010). Statins and the colorectum: Hope for chemoprevention?. Cancer Prev. Res..

[15] Ming M.E. (2011). The search for a chemoprevention agent effective against melanoma: Considerations and challenges. J. Investig. Dermatol..

[16] Pollak M. (2010). Metformin and other biguanides in oncology: Advancing the research agenda. Cancer Prev. Res..

[17] Shukla Y., George J. (2011). Combinatorial strategies employing nutraceuticals for cancer development. Ann. N. Y. Acad. Sci..

[18] Cragg G.M., Grothaus P.G., Newman D.J. (2009). Impact of natural products on developing new anti-cancer agents. Chem. Rev..

[19] Proksch P., Edrada R.A., Ebel R. (2002). Drugs from the seas—Current status and microbiological implications. Appl. Microbiol. Biotechnol..

[20] Tartar A., Boucias D.G., Becnel J.J., Adams B.J. (2003). Comparison of plastid 16S rRNA (rrn16) genes from *Helicosporidium* spp.: Evidence supporting the reclassification of Helicosporidia as green algae (Chlorophyta). Int. J. Syst. Evol. Microbiol..

[21] Ueno R., Urano N., Suzuki M. (2003). Phylogeny of the non photosynthetic green micro-algal genus Prototheca (Trebouxiophyceae, Chlorophyta) and related taxa inferred from SSU and LSU ribosomal DNA partial sequence data. FEMS Microbiol. Lett..

[22] Tartar A., Boucias D.G., Adams B.J., Becnel J.J. (2002). Phylogenetic analysis identifies the invertebrate pathogen *Helicosporidium* sp. as a green alga (Chlorophyta). Int. J. Syst. Evol. Microbiol..

[23] Irigoien X., Huisman J., Harris R.P. (2004). Global biodiversity patterns of marine phytoplankton and zooplankton. Nature.

[24] López-Urrutia A., San Martin E., Harris R.P., Irigoien X. (2006). Scaling the metabolic balance of the oceans. Proc. Natl. Acad. Sci. USA.

[25] Raff J.D., Njegic B., Chang W.L., Gordon M.S., Dabdub D., Gerber R.B., Finlayson-Pitts B.J. (2009). Chlorine activation indoors and outdoors via surface-mediated reactions of nitrogen oxides with hydrogen chloride. Proc. Natl. Acad. Sci. USA.

[26] Yaakob Z., Ali E., Zainal A., Mohamad M., Takriff M.S. (2014). An overview: Biomolecules from microalgae for animal feed and aquaculture. J. Biol. Res..

[27] Dominguez A., Ferreira M., Coutinho P., Fábregas J., Otero A. (2005). Delivery of astaxanthin from *Haematococcus pluvialis* to the aquaculture food chain. Aquaculture.

[28] Clarens A., Resurreccion E., White M., Colosi L. (2010). Environmental life cycle comparison of algae to other bioenergy feedstocks. Environ. Sci. Technol..

[29] Norsker N., Barbosa M., Vermue M., Wijffels R. (2011). Microalgal production-a close look at the economics. Biotechnol. Adv..

[30] Markou G., Iconomou D., Sotiroudis T., Israilides C., Muylaert K. (2015). Exploration of using stripped ammonia and ash from poultry litter for the cultivation of the cyanobacterium *Arthrospira platensis* and the green microalga *Chlorella vulgaris*. Bioresour. Technol..

[31] Kilian O., Benemann C., Niyogi K., Vick B. (2011). High-efficiency homologous recombination in the oil-producing alga *Nannochloropsis* sp. Proc. Natl. Acad. Sci. USA.

[32] Ahmad I., Sharma A.K., Daniell H., Kumar S. (2015). Altered lipid composition and enhanced lipid production in green microalga by introduction of brassica diacylglycerol acyltransferase 2. Plant Biotechnol. J..

[33] Yu X., Chen L., Zhang W. (2015). Chemicals to enhance microalgal growth and accumulation of high-value bioproducts. Front. Microbiol..

[34] Franz A.K., Danielewicz M.A., Wong D.M., Anderson L.A., Boothe J.R. (2013). Phenotypic screening with oleaginous microalgae reveals modulators of lipid productivity. ACS Chem. Biol..

[35] Sharma K., Schenk P.M. (2015). Rapid induction of omega-3 fatty acids (EPA) in *Nannochloropsis* sp. by UV-C radiation. Biotechnol. Bioeng..

[36] Jusoh M., Loh S.H., Chuah T.S., Aziz A., Cha T.S. (2015). Indole-3-acetic acid (IAA) induced changes in oil content, fatty acid profiles and expression of four fatty acid biosynthetic genes in *Chlorella vulgaris* at early stationary growth phase. Phytochemistry.

[37] Ramos A.A., Polle J.J., Tran D., Cushman J.C., Jin E., Varela J.C. (2011). The unicellular green alga *Dunaliella salina* Teod. as a model for abiotic stress tolerance: Genetic advances and future perspectives. Algae.

[38] Davidi L., Shimoni E., Khozin-Goldberg I., Zamir A., Pick U. (2014). Origin of b-carotene-rich plastoglobuli in *Dunaliella bardawil*. Plant Physiol..

[39] Yuan J.P., Peng J., Yin K., Wang J.H. (2011). Potential health-promoting effects of astaxanthin: A high-value carotenoid mostly from microalgae. Mol. Nutr. Food Res..

[40] Liu J., Sun Z., Gerken H., Liu Z., Jiang Y., Chen F. (2014). *Chlorella zofingiensis* as an alternative microalgal producer of astaxanthin: Biology and Industrial Potential. Mar. Drugs.

[41] Fu W., Gudmundsson O., Paglia G., Herjolfsson G., Andrésson O.S., Palsson B.Ø., Brynjolfsson S. (2013). Enhancement of carotenoid biosynthesis in the green microalga *Dunaliella salina* with light-emitting diodes and adaptive laboratory evolution. Appl. Microbiol. Biotechnol..

[42] Cordero B.F., Obraztsova I., Couso I., Leon R., Vargas M.A., Rodríguez H. (2011). Enhancement of lutein production in *Chlorella sorokiniana* (chlorophyta) by improvement of culture conditions and random mutagenesis. Mar. Drugs.

[43] Shi X.M., Chen F. (2002). High-yield production of lutein by the green microalga *Chlorella protothecoides* in heterotrophic fed-batch culture. Biotechnol. Prog..

[44] Pasquet V., Morisset P., Ihammouine S., Chepied A., Aumailley L., Berard J.-B., Serive B., Kaas R., Lanneluc I., Thiery V. (2011). Antiproliferative activity of violaxanthin isolated from bioguided fractionation of *Dunaliella tertiolecta* extracts. Mar. Drugs.

[45] Soontornchaiboon W., Joo S.S., Kim S.M. (2012). Anti-inflammatory effects of violaxanthin isolated from microalga *Chlorella ellipsoidea* in RAW 264.7 macrophages. Biol. Pharm. Bull..

[46] Lagarde D., Beuf L., Vermaas W. (2000). Increased production of zeaxanthin and other pigments by application of genetic engineering techniques to *Synechocystis* sp. strain PCC 6803. Appl. Environ. Microbiol..

[47] Singh D., Puri M., Wilkens S., Mathur A.S., Tuli D.K., Barrow C.J. (2013). Characterization of a new zeaxanthin producing strain of *Chlorella saccharophila* isolated from New Zealand marine waters. Bioresour. Technol..

[48] Kim Y., Seo J.H., Kim H. (2011). Beta-Carotene and lutein inhibit hydrogen peroxide-induced activation of NF-kappaB and IL-8 expression in gastric epithelial AGS cells. J. Nutr. Sci. Vitaminol..

[49] Crupi P., Toci A.T., Mangini S., Wrubl F., Rodolfi L., Tredici M.R., Coletta A., Antonacci D. (2013). Determination of fucoxanthin isomers in microalgae (*Isochrysis* sp.) by high-performance liquid chromatography coupled with diode-array detector multistage mass spectrometry coupled with positive electrospray ionization. Rapid Commun. Mass Spectrom..

[50] Mobraten K., Haug T.M., Kleiveland C.R., Lea T. (2013). Omega-3 and omega-6 PUFAs induce the same GPR120-mediated signalling events, but with different kinetics and intensity in Caco-2 cells. Lipids Health Dis..

[51] Adarme-Vega T.C., Thomas-Hall S.R., Lim D.K., Schenk P.M. (2014). Effects of long chain fatty acid synthesis and associated gene expression in microalga *Tetraselmis sp*. Mar. Drugs.

[52] Spencer L., Mann C., Metcalfe M., Webb M., Pollard C., Spencer D., Berry D., Steward W., Dennison A. (2009). The effect of omega-3 FAs on tumour angiogenesis and their therapeutic potential. Eur. J. Cancer.

[53] Lee I., Han J.I. (2015). Hydrothermal-acid treatment for effectual extraction of eicosapentaenoic acid (EPA)-abundant lipids from *Nannochloropsis salina*. Bioresour. Technol..

[54] Nauroth J.M., Liu Y.C., van Elswyk M., Bell R., Hall E.B., Chung G., Arterburn L.M. (2010). Docosahexaenoic acid (DHA) and docosapentaenoic acid (DPA*n*-6) algal oils reduce inflammatory mediators in human peripheral mononuclear cells *in vitro* and paw edema *in vivo*. Lipids.

[55] Maeda N., Kokai Y., Ohtani S., Sahara H., Kumamoto-Yonezawa Y., Kuriyama I., Hada T., Sato N., Yoshida H., Mizushina Y. (2008). Anti-tumor effect of orally administered spinach glycolipid fraction on implanted cancer cells, colon-26, in mice. Lipids.

[56] Mizushina Y., Hada T., Yoshida H. (2012). *In vivo* antitumor effect of liposomes with sialyl Lewis X including monogalactosyl diacylglycerol, a replicative DNA polymerase inhibitor, from spinach. Oncol. Rep..

[57] Hossain Z., Kurihara H., Hosokawa M., Takahashi K. (2005). Growth inhibition and induction of differentiation and apoptosis mediated by sodium butyrate in Caco-2 cells with algal glycolipids. In Vitro Cell Dev. Biol. Anim..

[58] Maeda N., Kokai Y., Ohtani S., Hada T., Yoshida H., Mizushina Y. (2009). Inhibitory effects of preventive and curative orally administered spinach glycoglycerolipid fraction on the tumor growth of sarcoma and colon in mouse graft models. Food Chem..

[59] Maeda N., Kokai Y., Hada T., Yoshida H., Mizushina Y. (2013). Oral administration of monogalactosyl diacylglycerol from spinach inhibits colon tumor growth in mice. Exp. Ther. Med..

[60] Guzmán S., Gato A., Lamela M., Freire-Garabal M., Calleja J.M. (2003). Anti-Inflammatory and immunomodulatory activities of polysaccharide from *Chlorella stigmatophora* and *Phaeodactylum tricornutum*. Phytother. Res..

[61] Nomoto K., Yokokura T., Satoh H., Mutai M. (1983). Anti-tumor effect by oral administration of Chlorella extract, PCM-4 by oral admission. Gan To Kagaku Zasshi.

[62] Jo W.S., Cho Y.J., Kim H.J., Nam B.Y., Hong S.H., Lee G.A., Lee S.W., Seo S.Y., Jeong M.H. (2010). Anti-inflammatory effect of microalgal extracts from *Tetraselmis suecica*. Food Sci. Biotechnol..

[63] Sadovskaya I., Souissi A., Souissi S., Grard T., Lencel P., Greene C.M., Duin S., Dmitrenok P.S., Chizhov A.O., Shashkov A.S. (2014). Chemical structure and biological activity of a highly branched (1→3,1→6)-β-d-glucan from *Isochrysis galbana*. Carbohydr. Polym..

[64] Matsui S.M., Muizzudin N., Arad S.M., Marenus K. (2003). Sulfated polysaccharides from red microalgae anti-inflammatory properties *in vitro* and *in vivo*. Appl. Biochem. Biotechnol..

[65] Bae S.Y., Yim J.H., Lee H.K., Pyo S. (2006). Activation of murine peritoneal macrophages by sulphated exopolysaccharide from marine microalga *Gyrodinium impudicum* (strain KG03): Involvement of the NF-kappa B and JNK pathway. Int. Immunopharmacol..

[66] Challouf R., Trabelsi L., Dhieb R.B., El Abed O., Yahia A., Ghozzi K., Ammar J.B., Omran H., Ouada H.B. (2011). Evaluation of cytotoxicity and biological activities in extracellular polysaccharides released by cyanobacterium *Arthrospira platensis*. Braz. Arch. Biol. Technol..

[67] Romay Ch., González R., Ledón N., Remirez D., Rimbau V. (2003). C-phycocyanin: A biliprotein with antioxidant, anti-inflammatory and neuroprotective effects. Curr. Protein Pept. Sci..

[68] Zheng L.H., Wang Y.J., Sheng J., Wang F., Zheng Y., Lin X.K., Sun M. (2011). Antitumor peptides from marine organisms. Mar. Drugs.

[69] Wang X., Zhang X. (2013). Separation, antitumor activities, and encapsulation of polypeptide from Chlorella pyrenoidosa. Biotechnol. Prog..

[70] Piplani H., Vaish V., Sanyal S.N. (2012). Dolastatin 15, a mollusk linear peptide, and Celecoxib, a selective cyclooxygenase-2 inhibitor, prevent preneoplastic colonic lesions and induce apoptosis through inhibition of the regulatory transcription factor NF-κB and an inflammatory protein, iNOS. Eur. J. Cancer Prev..

[71] Kwan J.C., Teplitski M., Gunasekera S.P., Paul V.J., Luesch H. (2010). Isolation and biological evaluation of 8-epi-malyngamide C from the Floridian marine cyanobacterium *Lyngbya majuscula*. J. Nat. Prod..

[72] Hatae N., Satoh R., Chiba H., Osaki T., Nishiyama T., Ishikura M., Abe T., Hibino S., Choshi T., Okada C. (2014). *N*-Substituted calothrixin B derivatives inhibited the proliferation of HL-60 promyelocytic leukemia cells. Med. Chem. Res..

[73] Abd El-Baky H.H., El-Baz F.K., El Baroty G.S. (2010). Enhancing antioxidant availability in wheat grains from plants grown under seawater stress in response to microalgae extract treatments. J. Sci. Food Agric..

[74] Cha T.S., Chen C.F., Yee W., Aziz A., Loh S.H. (2011). Cinnamic acid, coumarin and vanillin: Alternative phenolic compounds for efficient Agrobacterium-mediated transformation of the unicellular green alga, *Nannochloropsis* sp. J. Microbiol. Methods.

[75] Mendiola J.A., García-Martínez D., Ruperez F.J., Martín-Álvarez P.J., Reglero G., Cifuentes A., Barbas C., Ibañez E., Señoráns F.J. (2008). Enrichment of vitamin E from *Spirulina platensis* microalga by SFE. J. Supercrit. Fluid..

[76] DeSantis C.E., Lin C.C., Mariotto A.B., Siegel R.L., Stein K.D., Kramer J.L., Alteri R., Robbins A.S., Jemal A. (2014). Cancer treatment and survivorship statistics, 2014. CA Cancer J. Clin..

[77] O’Connor P.M., Lapointe T.K., Beck P.L., Buret A.G. (2010). Mechanisms by which inflammation may increase intestinal cancer risk in inflammatory bowel disease. Inflamm. Bowel Dis..

[78] Itzkowitz S.H., Yio X. (2004). Inflammation and cancer IV. Colorectal cancer in inflammatory bowel disease: The role of inflammation. Am. J. Physiol. Gastrointest. Liver Physiol..

[79] Lea M.A. (2010). Recently identified and potential targets for colon cancer treatment. Future Oncol..

[80] Talero E., Sánchez-Fidalgo S., Villegas I., de la Lastra C.A., Illanes M., Motilva V. (2011). Role of different inflammatory and tumor biomarkers in the development of ulcerative colitis-associated carcinogenesis. Inflamm. Bowel Dis..

[81] Riddle R.H., Goldman H., Ransohof D.F., Appelman H., Fenoglio C.M., Haggitt R. (1983). Dysplasia in inflammatory bowel disease: Standardized classification with provisional clinical application. Hum. Pathol..

[82] Pascal R.R. (1994). Dysplasia and early carcinoma in inflammatory bowel disease and colorectal carcinomas. Hum. Pathol..

[83] Bird P.R., Good C.K. (2000). The significance of aberrant crypt foci in understanding the pathogenesis of colon cancer. Toxico. Lett..

[84] Cooper H.S., Murthy S., Kido K., Yoshitake H., Flanigan A. (2000). Dysplasia and cancer in the dextran sulphate sodium mouse colitis model. Relevance to colitis-associated neoplasia in the human: A study of histopathology, β-catenin and expression and the role of inflammation. Carcinogenesis.

[85] Karin M. (2006). Nuclear factor-κB in cancer development and progression. Nature.

[86] Gustafsson A., Andersson M., Lagerstedt K., Lönnroth C., Nordgren S., Lundholm K. (2010). Receptor and enzyme expression for prostanoid metabolism in colorectal cancer related to tumor tissue PGE2. Int. J. Oncol..

[87] Rao S.K., Pavicevic Z., Du Z., Kim J.G., Fan M., Jiao Y., Rosebush M., Samant S., Gu W., Pfeffer L.M. (2010). Pro-inflammatory genes as biomarkers and therapeutic targets in oral squamous cell carcinoma. J. Biol. Chem..

[88] Aranda E., López-Pedrera C., Haba-Rodríguez J.R., Rodríguez-Ariza A. (2012). Nitric oxide and cancer: The emerging role of *S*-nitrosylation. Curr. Mol. Med..

[89] López-Lázaro M. (2009). Role of oxygen in cancer: Looking beyond hypoxia. Anticancer Agents Med. Chem..

[90] Scrivo R., Vasile M., Bartosiewicz I., Valesini G. (2011). Inflammation as “common soil” of the multifactorial diseases. Autoimmun. Rev..

[91] Yang C.A., Chiang B.L. (2015). Inflammasomes and human autoimmunity: A comprehensive review. J. Autoimmun..

[92] Han J.W., Shim D.W., Shin W.Y., Heo K.H., Kwak S.B., Sim E.J., Jeong J.H., Kang T.B., Lee K.H. (2015). Anti-inflammatory effect of emodin via attenuation of NLRP3 inflammasome activation. Int. J. Mol. Sci..

[93] Kim S.R., Kim D.I., Kim S.H., Lee H., Lee K.S., Cho S.H., Lee Y.C. (2014). NLRP3 inflammasome activation by mitochondrial ROS in bronchial epithelial cells is required for allergic inflammation. Cell Death Dis..

[94] Martinon F., Burns K., Tschopp J. (2002). The inflammasome: A molecular platform triggering activation of inflammatory caspases and processing of proIL-beta. Mol. Cell.

[95] Actis G.C. (2014). The gut microbiome. Inflamm. Allergy Drug Targets.

[96] Kanai T., Kamada N., Hisamatsu T. (2013). Clinical strategies for the blockade of IL-18 in inflammatory bowel diseases. Curr. Drug Targets.

[97] Chen G.Y., Núñez G. (2011). Inflammasomes in intestinal inflammation and cancer. Gastroenterology.

[98] Zaki M.H., Vogel P., Body-Malapel M., Lamkanfi M., Kanneganti T.D. (2010). IL-18 production downstream of the Nlrp3 inflammasome confers protection against colorectal tumor formation. J. Immunol..

[99] Saitoh T., Fujita N., Jang M.H., Uematsu S., Yang B.G., Satoh T., Omori H., Noda T., Yamamoto N., Komatsu M. (2008). Loss of the autophagy protein Atg16L1 enhances endotoxin-induced IL-1beta production. Nature.

[100] Zhou R., Yazdi A.S., Menu P., Tschopp J. (2011). A role for mitochondria in NLRP3 inflammasome activation. Nature.

[101] Salcedo R., Worschech A., Cardone M., Jones Y., Gyulai Z., Dai R.M., Wang E., Ma W., Haines D., O’hUigin C. (2010). MyD88-mediated signaling prevents development of adenocarcinomas of the colon: Role of interleukin 18. J. Exp. Med..

[102] Hu B., Elinav E., Flavell R.A. (2011). Inflammasome-mediated suppression of inflammation-induced colorectal cancer progression is mediated by direct regulation of epithelial cell proliferation. Cell Cycle.

[103] Allen I.C., TeKippe E.M., Woodford R.M., Uronis J.M., Holl E.K., Rogers A.B., Herfarth H.H., Jobin C., Ting J.P. (2010). The NLRP3 inflammasome functions as a negative regulator of tumorigenesis during colitis-associated cancer. J. Exp. Med..

[104] Williams T.M., Leeth R.A., Rothschild D.E., Coutermarsh-Ott S.L., McDaniel D.K., Simmons A.E., Heid B., Cecere T.E., Allen I.C. (2015). The NLRP1 inflammasome attenuates colitis and colitis-associated tumorigenesis. J. Immunol..

[105] Vaziri H., Dessain S.K., Ng Eaton E., Imai S.I., Frye R.A., Pandita T.K., Guarente L., Weinberg R.A. (2001). hSIR2 (SIRT1) functions as an NAD-dependent p53 deacetylase. Cell.

[106] Haigis M.C., Sinclair D.A. (2010). Mammalian sirtuins: Biological insights and disease relevance. Annu. Rev. Pathol..

[107] Kawahara T.L., Michishita E., Adler A.S., Damian M., Berber E., Lin M., McCord R.A., Ongaigui K.C., Boxer L.D., Chang H.Y. (2009). SIRT6 links histone H3 lysine 9 deacetylation to NF-kappaB-dependent gene expression and organismal life span. Cell.

[108] Cardus A., Uryga A.K., Walters G., Erusalimsky J.D. (2013). SIRT6 protects human endothelial cells from DNA damage, telomere dysfunction, and senescence. Cardiovasc. Res..

[109] Choi A.M., Ryter S.W., Levine B. (2013). Autophagy in human health and disease. N. Engl. J. Med..

[110] Hardie D.G. (2011). AMP-activated protein kinase: An energy sensor that regulates all aspects of cell function. Genes Dev..

[111] García-Mauriño S., Alcaide A., Domínguez C. (2012). Pharmacological control ofautophagy: Therapeutic perspectives in inflammatory bowel disease and colorectal cancer. Curr. Pharm. Des..

[112] Kato K., Ogura T., Kishimoto A., Minegishi Y., Nakajima N., Miyazaki M., Esumi H. (2002). Critical roles of AMP-activated protein kinase in constitutive tolerance of cancer cells to nutrient deprivation and tumor formation. Oncogene.

[113] Habeeb B.S., Kitayama J., Nagawa H. (2011). Adiponectin supports cell survival in glucose deprivation through enhancement of autophagic response in colorectal cancer cells. Cancer Sci..

[114] Koh S.J., Kim J.M., Kim I.K., Ko S.H., Kim J.S. (2014). Anti-inflammatory mechanism of metformin and its effects in intestinal inflammation and colitis-associated colon cancer. J. Gastroenterol. Hepatol..

[115] Firestein R., Blander G., Michan S., Oberdoerffer P., Ogino S., Campbell J., Bhimavarapu A., Luikenhuis S., de Cabo R., Fuchs C. (2008). The SIRT1 deacetylase suppresses intestinal tumorigenesis and colon cancer growth. PLoS ONE.

[116] Wang R.H., Sengupta K., Li C., Kim H.S., Cao L., Xiao C., Kim S., Xu X., Zheng Y., Chilton B. (2008). Impaired DNA damage response, genome instability, and tumorigenesis in SIRT1 mutant mice. Cancer Cell..

[117] Kabra N., Li Z., Chen L., Li B., Zhang X., Wang C., Yeatman T., Coppola D., Chen J. (2009). Sirt1 is an inhibitor of proliferation and tumor formation in colon cancer. J. Biol. Chem..

[118] Jang S.H., Min K.W., Paik S.S., Jang K.S. (2012). Loss of SIRT1 histone deacetylase expression associates with tumour progression in colorectal adenocarcinoma. J. Clin. Pathol..

[119] Kriegl L., Vieth M., Kirchner T., Menssen A. (2012). Up-regulation of c-MYC and SIRT1 expression correlates with malignant transformation in the serrated route to colorectal cancer. Oncotarget.

[120] Holloway K.R., Calhoun T.N., Saxena M., Metoyer C.F., Kandler E.F., Rivera C.A., Pruitt K. (2010). SIRT1 regulates Dishevelled proteins and promotes transient and constitutive Wnt signaling. Proc. Natl. Acad. Sci. USA.

[121] Etchegaray J.P., Zhong L., Mostoslavsky R. (2013). The histone deacetylase SIRT6: At the crossroads between epigenetics, metabolism and disease. Curr. Top Med. Chem..

[122] Cazzonelli C.I. (2011). Carotenoids in nature: Insights from plants and beyond. Func. Plant Biol..

[123] Guedes A.C., Amaro H.M., Malcata F.X. (2011). Microalgae as sources of carotenoids. Mar. Drugs.

[124] Begum H., Yusoff F.M., Banerjee S., Khatoon H., Shariff M. (2015). Availability and utilization of pigments from microalgae. Crit. Rev. Food Sci. Nutr..

[125] Varela J.C., Pereira H., Vila M., León R. (2015). Production of carotenoids by microalgae: Achievements and challenges. Photosynth. Res..

[126] Palozza P., Torelli C., Boninsegna A., Simone R., Catalano A., Mele M.C., Picci N. (2009). Growth-inhibitory effects of the astaxanthin-rich alga *Haematococcus pluvialis* in human colon cancer cells. Cancer Lett..

[127] Eonseon J., Polle J.E.W., Lee H.K., Hyund S.M., Chang M. (2003). Xanthophylls in microalgae: From biosynthesis to biotechnological mass production and application. Microb. Biotechnol..

[128] Brown M.R., Cruz-Suárez L.E., Ricque-Marie D., Tapia-Salazar M., Gaxiola-Cortés M.G., Simoes N. Nutritional value of microalgae for aquaculture. Advances en Nutrición Acuicola VI. Memorias del VI Simposium Internacional de Nutrición Acuicola, Cancun, Quintana Roo, Mexico 3–6 September 2002.

[129] Grune T., Lietz G., Palou A., Ross A.C., Stahl W., Tang G., Thurnham D., Yin S.A., Biesalski H.K. (2010). Beta-carotene is an important vitamin A source for humans. J. Nutr..

[130] Smith W., Saba N. (2005). Retinoids as chemoprevention for head and neck cancer: Where do we go from here?. Crit. Rev. Oncol. Hematol..

[131] Sun S.Y., Lotan R. (2002). Retinoids and their receptors in cancer development and chemoprevention. Crit. Rev. Oncol. Hematol..

[132] Lotan R. (1995). Retinoids and apoptosis: Implications for cancer chemoprevention and therapy. J. Natl. Cancer Inst..

[133] Ben-Amotz A., Mokady S., Avron M. (1988). The beta-carotene-rich alga Dunaliella bardawil as a source of retinol in a rat diet. Br. J. Nutr..

[134] Bhupathiraju S.N., Wedick N.M., Pan A., Manson J.E., Rexrode K.M., Willett W.C., Rimm E.B. (2013). Hu FB. Quantity and variety in fruit and vegetable intake and risk of coronary heart disease. Am. J. Clin. Nutr..

[135] Jansen R.J., Robinson D.P., Stolzenberg-Solomon R.Z., Bamlet W.R., de Andrade M., Oberg A.L., Rabe K.G., Anderson K.E., Olson J.E., Sinha R., Petersen G.M. (2013). Nutrients from fruit and vegetable consumption reduce the risk of pancreatic cancer. J. Gastrointest. Cancer.

[136] Kaulmann A., Serchi T., Renaut J., Hoffmann L., Bohn T. (2012). Carotenoid exposure of Caco-2 intestinal epithelial cells did not affect selected inflammatory markers but altered their proteomic response. Br. J. Nutr..

[137] Trivedi P.P., Jena G.B. (2015). Mechanistic insight into beta-carotene-mediated protection against ulcerative colitis-associated local and systemic damage in mice. Eur. J. Nutr..

[138] Lavy A., Naveh Y., Coleman R., Mokady S., Werman M.J. (2003). Dietary Dunaliella bardawil, a beta-carotene-rich alga, protects against acetic acid-induced small bowel inflammation in rats. Inflamm. Bowel Dis..

[139] Jowett S.L., Seal C.J., Pearce M.S., Phillips E., Gregory W., Barton J.R., Welfare M.R. (2004). Influence of dietary factors on the clinical course of ulcerative colitis: A prospective cohort study. Gut.

[140] Rumi G., Szabo I., Vincze A., Matus Z., Tóth G., Mózsik G. (2000). Decrease of serum carotenoids in Crohn’s disease. J. Physiol. Paris.

[141] Wendland B.E., Aghdassi E., Tam C., Carrrier J., Steinhart A.H., Wolman S.L., Baron D., Allard J.P. (2001). Lipid peroxidation and plasma antioxidant micronutrients in Crohn disease. Am. J. Clin. Nutr..

[142] Geerling B.J., Badart-Smook A., Stockbrügger R.W., Brummer R.J. (2000). Comprehensive nutritional status in recently diagnosed patients with inflammatory bowel disease compared with population controls. Eur. J. Clin. Nutr..

[143] Hengstermann S., Valentini L., Schaper L., Buning C., Koernicke T., Maritschnegg M., Buhner S., Tillinger W., Regano N., Guglielmi F. (2008). Altered status of antioxidant vitamins and fatty acids in patients with inactive inflammatory bowel disease. Clin. Nutr..

[144] Palozza P., Serini S., Maggiano N., Tringali G., Navarra P., Ranelletti F.O., Calviello G. (2005). Beta-carotene downregulates the steady-state and heregulin-alpha-induced COX-2 pathways in colon cancer cells. J. Nutr..

[145] Choi S.Y., Park J.H., Kim J.S., Kim M.K., Aruoma O.I., Sung M.K. (2006). Effects of quercetin and beta-carotene supplementation on azoxymethane-induced colon carcinogenesis and inflammatory responses in rats fed with high-fat diet rich in omega-6 fatty acids. Biofactors.

[146] Pham D.N.T., Leclerc D., Levesque N., Deng L., Rozen R. (2013). β,β-Carotene 15,15′-monooxygenase and its substrate β-carotene modulate migration and invasion in colorectal carcinoma cells. Am. J. Clin. Nutr..

[147] Raju J., Swamy M.V., Cooma I., Patlolla J.M., Pittman B., Reddy B.S., Steele V.E., Rao C.V. (2005). Low doses of beta-carotene and lutein inhibit AOM-induced rat colonic ACF formation but high doses augment ACF incidence. Int. J. Cancer.

[148] Mayne S.T. (1996). Beta-carotene, carotenoids, and disease prevention in humans. FASEB J..

[149] Mänistö S., Yaun S.S., Hunter D.J., Spiegelman D., Adami H.O., Albanes D., van den Brandt P.A., Buring J.E., Cerhan J.R., Colditz G.A. (2007). Dietary carotenoids and risk of colorectal cancer in a pooled analysis of 11 cohort studies. Am. J. Epidemiol..

[150] Wang Z., Joshi A.M., Ohnaka K., Morita M., Toyomura K., Kono S., Ueki T., Tanaka M., Kakeji Y., Maehara Y. (2012). Dietary Intakes of Retinol, Carotenes, Vitamin C, and Vitamin E and Colorectal Cancer Risk: The Fukuoka Colorectal Cancer Study. Nutr. Cancer.

[151] Leenders M., Leufkens A.M., Siersema P.D., van Duijnhoven F.J., Vrieling A., Hulshof P.J., van Gils C.H., Overvad K., Roswall N., Kyrø C. (2014). Plasma and dietary carotenoids and vitamins A, C and E and risk of colon and rectal cancer in the European Prospective Investigation into Cancer and Nutrition. Int. J. Cancer.

[152] Brown G.T., Cash B.G., Blihoghe D., Johansson P., Alnabulsi A., Murray G.I. (2014). The expression and prognostic significance of retinoic acid metabolising enzymes in colorectal cancer. PLoS ONE.

[153] Kropotova E.S., Zinovieva O.L., Zyryanova A.F., Dybovaya V.I., Prasolov V.S., Beresten S.F., Oparina N.Y., Mashkova T.D. (2014). Altered expression of multiple genes involved in retinoic acid biosynthesis in human colorectal cancer. Pathol. Oncol. Res..

[154] Chaiter Y., Gruber S.B., Ben-Amotz A., Almog R., Rennert H.S., Fischler R., Rozen G., Rennert G. (2009). Smoking attenuates the negative association between carotenoids consumption and colorectal cancer risk. Cancer Causes Control.

[155] Kabat G.C., Kim M.Y., Sarto G.E., Shikany J.M., Rohan T.E. (2012). Repeated measurements of serum carotenoid, retinol and tocopherol levels in relation to colorectal cancer risk in the Women’s Health Initiative. Eur. J. Clin. Nutr..

[156] Jung S., Wu K., Giovannucci E., Spiegelman D., Willett W.C., Smith-Warner S.A. (2013). Carotenoid intake and risk of colorectal adenomas in a cohort of male health professionals. Cancer Causes Control.

[157] Lu M.S., Fang Y.J., Chen Y.M., Luo W.P., Pan Z.Z., Zhong X., Zhang C.X. (2015). Higher intake of carotenoid is associated with a lower risk of colorectal cancer in Chinese adults: A case-control study. Eur. J. Nutr..

[158] Johnson E.A., An G.H. (1991). Astaxanthin from microbial sources. Crit. Rev. Biotechnol..

[159] Jyonouchi H., Sun S., Gross M. (1995). Astaxanthin, a carotenoid without vitamin A activity, augments antibody responses in cultures including T-helper cell clones and suboptimal doses of antigen. J. Nutr..

[160] Barros M.P., Poppe S.C., Bondan E.F. (2014). Neuroprotective properties of the marine carotenoidastaxanthin and omega-3 fatty acids, and perspectives for the natural combination of both in krill oil. Nutrients.

[161] Choi S.K., Park Y.S., Choi D.K., Chang H.I. (2008). Effects of astaxanthin on the production of NO and the epression of COX-2 and iNOS in LPS-simulated BV2 microglial cells. J. Microbiol. Biotechnol..

[162] Tanaka T., Kawamori T., Ohnishi M., Makita H., Mori H., Satoh K., Hara A. (1995). Suppression of azomethane-induced rat colon carcinogenesis by dietary administration of naturally occurring xanthophylls astaxanthin and canthaxanthin during the postinitiation phase. Carcinogenesis.

[163] Prabhu P.N., Ashokkumar P., Sudhandiran G. (2009). Antioxidative and antiproliferative effects of astaxanthin during the initiation stages of 1,2-dimethyl hydrazineinduced experimental colon carcinogenesis. Fund. Clin. Pharmacol..

[164] Nagendraprabhu P., Sudhandiran G. (2011). Astaxanthin inhibits tumor invasion by decreasing extracellular matrix production and induces apoptosis in experimental rat colon carcinogenesis by modulating the expressions of ERK-2, NFkB and COX-2. Investig. New Drugs.

[165] Yasui Y., Hosokawa M., Mikami N., Miyashita K., Tanaka T. (2011). Dietary astaxanthin inhibits colitis and colitis-associated colon carcinogenesis in mice via modulation of the inflammatory cytokines. Chem. Biol. Interact..

[166] Kupcinskas L., Lafolie P., Lignell A., Kiudelis G., Jonaitis L., Adamonis K., Andersen L.P., Wadström T. (2008). Efficacy of the natural antioxidant astaxanthin in the treatment of functional dyspepsia in patients with or without *Helicobacter pylori* infection: A prospective, randomized, double blind, and placebo-controlled study. Phytomedicine.

[167] Park J.S., Chyun J.H., Kim Y.K., Line L.L., Chew B.P. (2010). Astaxanthin decreased oxidative stress and inflammation and enhanced immune response in humans. Nutr. Metab. (Lond.).

[168] Yoon H.S., Cho H.H., Cho S., Lee S.R., Shin M.H., Chung J.H. (2014). Supplementating with dietary astaxanthin combined with collagen hydrolysate improves facial elasticity and decreases matrix metalloproteinase-1 and -12 expression: A comparative study with placebo. J. Med. Food.

[169] Fernandez-Sevilla J.M., Acien Fernandez F.G., Molina Grima E. (2010). Biotechnological production of lutein and its applications. Appl. Microbiol. Biotechnol..

[170] Carpentier S., Knaus M., Suh M. (2009). Associations between lutein, zeaxanthin, and age-related macular degeneration: An overview. Crit. Rev. Food Sci. Nutr..

[171] Lin J.H., Lee D.J., Chang J.S. (2015). Lutein production from biomass: Marigold flowers *versus* microalgae. Bioresour. Technol..

[172] Jahns P., Holzwarth A.R. (2012). The role of the xanthophyll cycle and of lutein in photoprotection of photosystem II. Biochim. Biophys. Acta.

[173] Nidhi B., Sharavana G., Ramaprasada T.R., Vallikannan B. (2015). Lutein derived fragments exhibit higher antioxidant and anti-inflammatory properties than lutein in lipopolysaccharide induced inflammation in rats. Food Funct..

[174] Krishnaswamy R., Devaraj S.N., Padma V.V. (2010). Lutein protects HT-29 cells against Deoxynivalenol-induced oxidative stress and apoptosis: Prevention of NF-kappaB nuclear localization and down regulation of NF-kappaB and Cyclo-Oxygenase-2 expression. Free Radic. Biol. Med..

[175] Murtaugh M.A., Ma K.N., Caan B.J., Sweeney C., Wolff R., Samowitz W.S., Potter J.D., Slattery M.L. (2005). Interactions of peroxisome proliferator-activated receptor {gamma} and diet in etiology of colorectal cancer. Cancer Epidemiol. Biomarkers Prev..

[176] Cha K.H., Koo S.Y., Lee D.U. (2008). Antiproliferative effects of carotenoids extracted from Chlorella ellipsoidea and Chlorella vulgaris on human colon cancer cells. J. Agric. Food Chem..

[177] Reynoso-Camacho R., González-Jasso E., Ferriz-Martínez R., Villalón-Corona B., Loarca-Piña G.F., Salgado L.M., Ramos-Gomez M. (2011). Dietary supplementation of lutein reduces colon carcinogenesis in DMH-treated rats by modulating K-ras, PKB, and β-catenin proteins. Nutr. Cancer.

[178] Bian Q., Gao S., Zhou J., Qin J., Taylor A., Johnson E.J., Tang G., Sparrow J.R., Gierhart D., Shang F. (2012). Lutein and zeaxanthin supplementation reduces photooxidative damage and modulates the expression of inflammation-related genes in retinal pigment epithelial cells. Free Radic. Biol. Med..

[179] Chen F., Li H.B., Wong R.N., Ji B., Jiang Y. (2005). Isolation and purification of the bioactive carotenoid zeaxanthin from the microalga *Microcystis aeruginosa* by high-speed counter-current chromatography. J. Chromatogr. A..

[180] Chen C.R., Hong S.E., Wang Y.C., Hsu S.L., Hsiang D., Chang C.M. (2012). Preparation of highly pure zeaxanthin particles from sea water-cultivated microalgae using supercritical anti-solvent recrystallization. Bioresour. Technol..

[181] Bian Q., Qin T., Ren Z., Wu D., Shang F. (2012). Lutein or zeaxanthin supplementation suppresses inflammatory responses in retinal pigment epithelial cells and macrophages. Adv. Exp. Med. Biol..

[182] Okuyama Y., Ozasa K., Oki K., Nishino H., Fujimoto S., Watanabe Y. (2014). Inverse associations between serum concentrations of zeaxanthin and other carotenoids and colorectal neoplasm in Japanese. Int. J. Clin. Oncol..

[183] Peng J., Yuan J.P., Wu C.F., Wang J.H. (2011). Fucoxanthin, a marine carotenoid present in brown seaweeds and diatoms: Metabolism and bioactivities relevant to human health. Mar. Drugs.

[184] Gagez A.L., Thiery V., Pasquet V., Cadoret J.P., Picot L. (2012). Epoxycarotenoids and cancer. Review. Curr. Bioact. Compd..

[185] Maeda H., Masashi Hosokawa M., Sashima T., Funayama K., Miyashita K. (2005). Fucoxanthin from edible seaweed, *Undaria pinnatifida*, shows antiobesity effect through UCP1 expression in white adipose tissues. Biochem. Biophys. Res. Commun..

[186] Kumar S.R., Hosokawa M., Miyashita K. (2013). Fucoxanthin: A marine carotenoid exerting anti-cancer effects by affecting multiple mechanisms. Mar. Drugs.

[187] Hosokawa M., Kudo M., Maeda H., Kohno H., Tanaka T., Miyashita K. (2004). Fucoxanthin induces apoptosis and enhances the antiproliferative effect of the PPARγ ligand, troglitazone, on colon cancer cells. Biochim. Biophys. Acta.

[188] Kawee-Ai A., Kim S.M. (2014). Application of microalgal fucoxanthin for the reduction of colon cancer risk: Inhibitory activity of fucoxanthin against beta-glucuronidase and DLD-1 cancer cells. Nat. Prod. Commun..

[189] Yates C.M., Calder P.C., Ed Rainger G. (2014). Pharmacology and therapeutics of omega-3 polyunsaturated fatty acids in chronic inflammatory disease. Pharmacol. Ther..

[190] Augustsson K., Michaud D.S., Rimm E.B., Leitzmann M.F., Stampfer M.J., Willett W.C., Giovannucci E. (2003). A prospective study of intake of fish and marine fatty acids and prostate cancer. Cancer Epidemiol. Biomarkers Prev..

[191] Cheng J., Ogawa K., Kuriki K., Yokoyama Y., Kamiya T., Seno K., Okuyama H., Wang J., Luo C., Fujii T. (2003). Increased intake of *n*-3 poly (DPA)unsaturated fatty acids elevates the level of apoptosis in the normal sigmoid colon of patients polypectomized for adenomas/tumors. Cancer Lett..

[192] Dommels Y.E., Haring M.M., Keestra N.G., Alink G.M., van Bladeren P.J., van Ommen B. (2003). The role of cyclooxygenase in *n*-6 and *n*-3 polyunsaturated fatty acid mediated effects on cell proliferation, PGE(2) synthesis and cytotoxicity in human colorectal carcinoma cell lines. Carcinogenesis.

[193] Feagan B.G., Sandborn W.J., Mittmann U., Bar-Meir S., D’Haens G., Bradette M., Cohen A., Dallaire C., Ponich T.P., McDonald J.W. (2008). Omega-3 free fatty acids for the maintenance of remission in Crohn disease: The EPIC Randomized Controlled Trials. JAMA.

[194] Serini S., Bizzarro A., Piccioni E., Fasano E., Rossi C., Lauria A., Cittadini A.R., Masullo C., Calviello G. (2012). EPA and DHA differentially affect *in vitro* inflammatory cytokine release by peripheral blood mononuclear cells from Alzheimer’s patients. Curr. Alzheimer Res..

[195] Das U.N. (2011). Can vagus nerve stimulation halt or ameliorate rheumatoid arthritis and lupus?. Lipids Health Dis..

[196] Bosco N., Brahmbhatt V., Oliveira M., Martin F.P., Lichti P., Raymond F., Mansourian R., Metairon S., Pace-Asciak C., Bastic Schmid V. (2013). Effects of increase in fish oil intake on intestinal eicosanoids and inflammation in a mouse model of colitis. Lipids Health Dis..

[197] Mobraten K., Haugbro T., Karlstrom E., Kleiveland C.R., Lea T. (2014). Activation of the bile acid receptor TGR5 enhances LPS-induced inflammatory responses in a human monocytic cell line. J Recept Signal Transduct Res..

[198] Van Beelen V.A., Spenkelink B., Mooibroek H., Sijtsma L., Bosch D., Rietjens I.M., Alink G.M. (2009). An n-3 PUFA-rich microalgal oil diet protects to a similar extent as a fish oil-rich diet against AOM-induced colonic aberrant crypt foci in F344 rats. Food Chem. Toxicol..

[199] Wall R., Ross R.P., Fitzgerald G.F., Stanton C. (2010). Fatty acids from fish: The anti-inflammatory potential of long-chain omega-3 fatty acids. Nutr Rev..

[200] Yurko-Mauro K., Kralovec J., Bailey-Hall E., Smeberg V., Stark J.G., Salem N. (2015). Similar eicosapentaenoic acid and docosahexaenoic acid plasma levels achieved with fish oil or krill oil in a randomized double-blind four-week bioavailability study. Lipids Health Dis..

[201] Ibrahim A., Mbodji K., Hassan A., Aziz M., Boukhettala N., Coëffier M., Savoye G., Déchelotte P., Marion-Letellier R. (2011). Anti-inflammatory and anti-angiogenic effect of long chain *n*-3 polyunsaturated fatty acids in intestinal microvascular endothelium. Clin. Nutr..

[202] Lee T.H., Hoover R.L., Williams J.D., Sperling R.I., Ravalese J., Spur B.W., Robinson D.R., Corey E.J., Lewis R.A., Austen K.F. (1985). Effects of dietary enrichment with eicosapentaenoic acid and docosahexaenoic acid on *in vitro* neutrophil and monocyte leukotriene generation and neutrophil function. N. Eng. J. Med..

[203] Rees D., Miles E.A., Banerjee T., Wells S.J., Roynette C.E., Wahle K.W., Calder P.C. (2006). Dose-related effects of eicosapentaenoic acid on innate immune function in healthy humans: A comparison of young and older men. Am. J. Clin. Nutr..

[204] Sun Y.P., Tjonahen E., Keledjian R., Zhu M., Yang R., Recchiuti A., Pillai P.S., Petasis N.A., Serhan C.N. (2009). Anti-inflammatory and pro-resolving properties of benzo-lipoxin A4 analogs. Prostaglandins Leukot. Essent. Fatty Acids.

[205] Serhan C.N., Hong S., Gronert K., Colgan S.P., Devchand P.R., Mirick G., Moussignac R.L. (2002). Resolvins: A family of bioactive products of omega-3 fatty acid transformation circuits initiated by aspirin treatment that counter proinflammation signals. J. Exp. Med..

[206] De Los Reyes C., Ávila-Román J., Ortega M.J., de la Jara A., García-Mauriño S., Motilva V., Zubía E. (2014). Oxylipins from the microalgae *Chlamydomonas debaryana* and *Nannochloropsis gaditana* and their activity as TNF-α inhibitors. Phytochemistry.

[207] Avila-Román J., Talero E., Alcaide A., de los Reyes C., Zubía E., García-Mauriño S., Motilva V. (2014). Preventive effect of the microalga *Chlamydomonas debaryana* on the acute phase of experimental colitis in rats. Br. J. Nutr..

[208] Colombo D., Gagliardi C., Vetro M., Ronchetti F., Takasaki M., Konoshima T., Suzuki N., Tokuda H. (2013). New 6-amino-6-deoxy-glycoglycerolipids derived from 2-*O*-b-d-glu-copyranosylglycerol: Insights into the structure–activity relationship of glycoglycerolipids as anti-tumor promoters. Carbohydr. Res..

[209] Mercurio A.M., Schwarting G.A., Robbins P.W. (1984). Glycolipids of the mouse peritoneal macrophage. Alterations in amount and surface exposure of specific glycolipid species occur in response to inflammation and tumoricidal activation. J. Exp. Med..

[210] Parrish C.C., Bodennec G., Gentien P. (1998). Haemolytic glycoglycerolipids from Gymnodinium species. Phytochemistry.

[211] Meireles L.A., Guedes A.C., Malcata F.X. (2003). Lipid class composition of the microalga *Pavlova lutheri*: Eicosapentaenoic and docosahexaenoic acids. J. Agric. Food Chem..

[212] Xu J., Chen D., Yan X., Chen J., Zhou C. (2010). Global characterization of the photosynthetic glycerolipids from a marine diatom *Stephanodiscus* sp. by ultra performance liquid chromatography coupled with electrospray ionization-quadrupole-time of flight mass spectrometry. Anal. Chim. Acta.

[213] MacDougall K.M., McNichol J., McGinn P.J., O’Leary S.J., Melanson J.E. (2011). Triacylglycerol profiling of microalgae strains for biofuel feedstock by liquid chromatography-high-resolution mass spectrometry. Anal. Bioanal. Chem..

[214] Yamane K., Matsuyama S., Igarashi K., Utsumi M., Shiraiwa Y., Kuwabara T. (2013). Anaerobic coculture of microalgae with *Thermosipho globiformans* and *Methanocaldococcus jannaschii* at 68 °C enhances generation of *n*-alkane-rich biofuels after pyrolysis. Appl. Environ. Microbiol..

[215] Banskota A.H., Gallant P., Stefanova R., Melanson R., O’Leary S.J. (2013). Monogalactosyldiacylglycerols, potent nitric oxide inhibitors from the marine microalga *Tetraselmis chui*. Nat. Prod. Res..

[216] Banskota A.H., Stefanova R., Sperker S., Lall S.P., Craigie J.S., Hafting J.T., Critchley A.T. (2014). Polar lipids from the marine macroalga *Palmaria palmata* inhibit lipopolysaccharide-induced nitric oxide production in RAW264.7 macrophage cells. Phytochemistry.

[217] Maeda N., Hada T., Murakami-Nakai C., Kuriyama I., Ichikawa H., Fukumori Y., Hiratsuka J., Yoshida H., Sakaguchi K., Mizushina Y. (2005). Effects of DNA polymerase inhibitory and antitumor activities of lipase-hydrolyzed glycolipid fractions from spinach. J. Nutr. Biochem..

[218] Kagan M.L., Levy A., Leikin-Frenkel A. (2015). Comparative study of tissue deposition of omega-3 fatty acids from polar-lipid rich oil of the microalgae *Nannochloropsis oculata* with krill oil in rats. Food Funct..

[219] De Jesús Raposo M.F., de Morais A.M.M.B., de Morais R.M.S.C. (2015). Marine polysaccharides from algae with potential biomedical applications. Mar. Drugs.

[220] De Jesus Raposo M.F., de Morais A.M.M.B., de Morais R.M.S.C., Merillon J.M., Ramawat K.G. (2014). Bioactivity and Applications of polysaccharides from marine microalgae. Polysaccharides: Bioactivity and Biotechnology.

[221] Sun Y., Wang H., Guo G., Pu Y., Yan B. (2014). The isolation and antioxidant activity of polysaccharides from the marine microalgae *Isochrysis galbana*. Carbohydr. Polym..

[222] Sun L., Wang L., Li J., Liu H. (2014). Characterization and antioxidant activities of degraded polysaccharides from two marine Chrysophyta. Food Chem..

[223] Miao X.P., Sun X.N., Cui L.J., Cao Q.F., Zhuang G.F., Deng T.Z., Zhang D.Y. (2015). Suppressive effect of pectic polysaccharides extracted from *Rauwolfia verticillata* (Lour.) Baill.var.hainanensis Tsiang on inflammation by regulation of NF-κB pathway and interleukin-17 in mice with dextran sulphatesodium-induced ulcerative colitis. Asian Pac. J. Trop. Med..

[224] Hartog A., Belle F.N., Bastiaans J., de Graaff P., Garssen J., Harthoorn L.F., Vos A.P. (2015). A potential role for regulatory T-cells in the amelioration of DSS induced colitis by dietary non-digestible polysaccharides. J. Nutr. Biochem..

[225] Umemura K., Yanase K., Suzuki M., Okutani K., Yamori T., Andoh T. (2003). Inhibition of DNA topoisomerases I and II, and growth inhibition of human cancer cell lines by a marine microalgal polysaccharide. Biochem. Pharm..

[226] Pugh N., Ross S.A., ElSohly H.N., ElSohly M.A., Pasco D.S. (2001). Isolation of three high molecular weight polysaccharide preparations with potent immunostimulatory activity from *Spirulina platensis*, *Aphanizomenon flos-aquae* and *Chlorella pyrenoidosa*. Planta Med..

[227] Balachandran P., Pugh N.D., Ma G., Pasco D.S. (2006). Toll-like receptor 2-dependent activation of monocytes by *Spirulina* polysaccharide and its immune enhancing action in mice. Int Immunopharmacol..

[228] Hayashi O., Ishii K., Kawamura C., YenHei S., YeBao N., Hirahashi T., Katoh T. (2004). Enhancement of mucosal immune functions by dietary *Spirulina plantensis* in human and animals. Nutr. Sci..

[229] Samarakoon K., Jeon Y.J. (2012). Bio-functionalities of proteins derived from marine algae—A review. Food Res. Int..

[230] Rasmussen R.S., Morrissey M.T. (2007). Marine biotechnology for production of food ingredients. Adv. Food Nutr. Res..

[231] Li B., Gao M.H., Zhang X.C., Chu X.M. (2006). Molecular immune mechanism of C-phycocyanin from *Spirulina platensis* induces apoptosis in HeLa cells *in vitro*. Biotechnol. Appl. Biochem..

[232] Saini M.K., Sanyal S.N., Vaiphei K. (2012). Piroxicam and C-phycocyanin mediated apoptosis in 1,2-dimethylhydrazine dihydrochloride induced colon carcinogenesis: Exploring the mitochondrial pathway. Nutr. Cancer.

[233] Kim J.A., Kim S.K. (2013). Bioactive peptides from marine sources as potential anti-inflammatory therapeutics. Curr. Protein Pept. Sci..

[234] Kang K.H., Kim S.K. (2013). Beneficial effect of peptides from microalgae on anticancer. Curr. Protein Pept. Sci..

[235] Suetsuna K., Chen J.R. (2001). Identification of antihypertensive peptides from peptic digests of two microalgae, *Chlorella vulgaris* and *Spirulina platensis*. Mar. Biotechnol..

[236] Linington R.G., Edwards D.J., Shuman C.F., McPhail K.L., Matainaho T., Gerwick W.H. (2008). Symplocamide A, a potent cytotoxin and chymotrypsin inhibitor from the marine cyanobacterium *Symploca* sp. J. Nat. Prod..

[237] Wrasidlo W., Mielgo A., Torres V.A., Barbero S., Stoletov K., Suyama T.L., Klemke R.L., Gerwick W.H., Carson D.A., Stupack D.G. (2008). The marine lipopeptide somocystinamide A triggers apoptosis via caspase 8. Proc. Natl. Acad. Sci. USA.

[238] Gutierrez M., Suyama T.L., Engene N., Wingerd J.S., Matainaho T., Gerwick W.H. (2008). Apratoxin D, a potent cytotoxic cyclodepsipeptide from papua new guinea collections of the marine cyanobacteria *Lyngbya majuscula* and *Lyngbya sordida*. J. Nat. Prod..

[239] Gunasekera S.P., Ross C., Paul V.J., Matthew S., Luesch H. (2008). Dragonamides C and D, linear lipopeptides from the marine cyanobacterium brown *Lyngbya polychroa*. J. Nat. Prod..

[240] Andrianasolo E.H., Goeger D., Gerwick W.H. (2007). Mitsoamide: A cytotoxic linear lipopeptide from the Madagascar marine cyanobacterium *Geitlerinema* sp. Pure Appl. Chem..

[241] Chaganty S., Golakoti T., Heltzel C., Moore R.E., Yoshida W.Y. (2004). Isolation and structure determination of cryptophycins 38, 326, and 327 from the terrestrial cyanobacterium *Nostoc* sp. GSV 224. J. Nat. Prod..

[242] Subramanian B., Nakeff A.E., Media J.E., Wiegand R.A., Valeriote F.A. (2002). Inhibition of macromolecular synthesis by cryptophycin-52. Anticancer Drugs.

[243] Simmons T.L., Nogle L.M., Media J., Valeriote F.A., Mooberry S.L., Gerwick W.H. (2009). Desmethoxymajusculamide C, a cyanobacterial depsipeptide with potent cytotoxicity in both cyclic and ring-opened forms. J. Nat. Prod..

[244] Mooberry S.L., Leal R.M., Tinley T.L., Luesch H., Moore R.E., Corbett T.H. (2003). The molecular pharmacology of symplostatin 1: A new antimitotic dolastatin 10 analog. Int. J. Cancer.

[245] Pereira A.R., Kale A.J., Fenley A.T., Byrum T., Debonsi H.M., Gilson M.K., Valeriote F.A., Moore B.S., Gerwick W.H. (2012). The carmaphycins: New proteasome inhibitors exhibiting an α,β-epoxyketone warhead from a marine cyanobacterium. Chembiochem.

[246] Kang H.S., Krunic A., Shen Q., Swanson S.M., Orjala J. (2011). Minutissamides A–D, antiproliferative cyclic decapeptides from the cultured cyanobacterium *Anabaena minutissima*. J. Nat. Prod..

[247] Montaser R., Paul V.J., Luesch H. (2011). Pitipeptolides C–F, antimycobacterial cyclodepsipeptides from the marine cyanobacterium *Lyngbya majuscula* from Guam. Phytochemistry.

[248] Liu H., Liu Y., Wang Z., Xing X., Maguire A. R., Luesch H., Zhang H., Xu Z., Ye T. (2013). Total synthesis and biological evaluation of Grassypeptolide A. Chem. Eur. J..

[249] Fennell B.J., Carolan S., Pettit G.R., Bell A. (2003). Effects of the antimitotic natural product dolastatin 10, and related peptides, on the human malarial parasite *Plasmodium falciparum*. J. Antimicrob. Chemother..

[250] Aherne G.W., Hardcastle A., Valenti M., Bryant A., Rogers P., Pettit G.R., Srirangam J.K., Kelland L.R. (1996). Antitumour evaluation of dolastatins 10 and 15 and their measurement in plasma by radioimmunoassay. Cancer Chemother. Pharmacol..

[251] Kobayashi M., Natsume T., Tamaoki S., Watanabe J., Asano H., Mikami T., Miyasaka K., Miyazaki K., Gondo M., Sakakibara K. (1997). Antitumor activity of TZT-1027, a novel dolastatin 10 derivative. Jpn. J. Cancer Res..

[252] Fujita F., Koike M., Fujita M., Sakamoto Y., Tsukagoshi S. (2000). Antitumor effects of TZT-1027, a novel dolastatin 10 derivative, on human tumor xenografts in nude mice. Gan To Kagaku Ryoho..

[253] Shnyder S.D., Cooper P.A., Millington N.J., Pettit G.R., Bibby M.C. (2007). Auristatin PYE, a novel synthetic derivative of dolastatin 10, is highly effective in human colon tumour models. Int. J. Oncol..

[254] Prokopiou E.M., Cooper P.A., Pettit G.R., Bibby M.C., Shnyder S.D. (2010). Potentiation of the activity of cisplatin in a human colon tumour xenograft model by auristatin PYE, a structural modification of dolastatin 10. Mol. Med. Rep..

[255] Piplani H., Rana C., Vaish V., Vaiphei K., Sanyal S.N. (2013). Dolastatin, along with Celecoxib, stimulates apoptosis by a mechanism involving oxidative stress, membrane potential change and PI3-K/AKT pathway down regulation. Biochim. Biophys. Acta.

[256] Salvador-Reyes L.A., Engene N., Paul V.J., Luesch H. (2015). Targeted natural products discovery from marine cyanobacteria using combined phylogenetic and mass spectrometric evaluation. J. Nat. Prod..

[257] Uzair B., Tabassum S., Rasheed M., Rehman S.F. (2012). Exploring marine cyanobacteria for lead compounds of pharmaceutical importance. Sci. World J..

[258] Bernardo P.H., Chai C.L., Heath G.A., Mahon P.J., Smith G.D., Waring P., Wilkes B.A. (2004). Synthesis, electrochemistry, and bioactivity of the cyanobacterial calothrixins and related quinones. J. Med. Chem..

[259] Kang H.S., Santarsiero B.D., Kim H., Krunic A., Shen Q., Swanson S.M., Chai H., Kinghorn A.D., Orjala J. (2012). Merocyclophanes A and B, antiproliferative cyclophanes from the cultured terrestrial Cyanobacterium *Nostoc* sp. Phytochemistry.

[260] Leaõ P.N., Costa M., Ramos V., Pereira A.R., Fernandes V.C., Domingues V.F., Gerwick W.H., Vasconcelos V.M., Martins R. (2013). Antitumor activity of Hierridin B, a cyanobacterial secondary metabolite found in both filamentous and unicellular marine strains. PLoS ONE.

[261] Ramos-e-Silva M., Jacques C. (2012). Epidermal barrier function and systemic diseases. Clin. Dermatol..

[262] Grether-Beck S., Marini A., Jaenicke T., Krutmann J. (2014). Photoprotection of human skin beyond ultraviolet radiation. Photodermatol. Photoimmunol. Photomed..

[263] Battie C., Jitsukawa S., Bernerd F., del Bino S., Marionnet C., Verschoore M. (2014). New insights in photoaging, UVA induced damage and skin types. Exp. Dermatol..

[264] Mancebo S.E., Wang S.Q. (2014). Skin cancer: Role of ultraviolet radiation in carcinogenesis. Rev. Environ. Health.

[265] Chen A.C., Halliday G.M., Damian D.L. (2013). Non-melanoma skin cancer: Carcinogenesis and chemoprevention. Pathology.

[266] Gordon R. (2013). Skin cancer: An overview of epidemiology and risk factors. Semin. Oncol. Nurs..

[267] McCusker M., Basset-Seguin N., Dummer R., Lewis K., Schadendorf D., Sekulic A., Hou J., Wang L., Yue H., Hauschild A. (2014). Metastatic basal cell carcinoma: Prognosis dependent on anatomic site and spread of disease. Eur. J. Cancer.

[268] Berwick M., Pestak C., Thomas N. (2014). Solar ultraviolet exposure and mortality from skin tumors. Adv. Exp. Med. Biol..

[269] Wells J.W. (2015). Do actinic keratoses and superficial squamous cell carcinomas have a specific immunoprofile?. Curr. Probl. Dermatol..

[270] Goldenberg G., Perl M. (2014). Actinic keratosis: Update on field therapy. J. Clin. Aesthet. Dermatol..

[271] Kaskel P., Lange U., Sander S., Huber M.A., Utikal J., Leiter U., Krahn G., Meurer M., Kron M. (2015). Ultraviolet exposure and risk of melanoma and basal cell carcinoma in Ulm and Dresden, Germany. J. Eur. Acad. Dermatol. Venereol..

[272] Werner R.N., Sammain A., Erdmann R., Hartmann V., Stockfleth E., Nast A. (2013). The natural history of actinic keratosis: A systematic review. Br. J. Dermatol..

[273] Wheller L., Soyer H.P. (2015). Clinical features of actinic keratoses and early squamous cell carcinoma. Curr. Probl. Dermatol..

[274] Chetty P., Choi F., Mitchell T. (2015). Primary care review of actinic keratosis and its therapeutic options: A global perspective. Dermatol. Ther..

[275] Singh M., Suman S., Shukla Y. (2014). New enlightenment of skin cancer chemoprevention through phytochemicals: *In vitro* and *in vivo* studies and the underlying mechanisms. Biomed. Res. Int..

[276] Nindl I., Gottschling M., Krawtchenko N., Lehmann M.D., Rowert-Huber J., Eberle J., Stockfleth E., Forschner T. (2007). Low prevalence of p53, p16(INK4a) and Ha-ras tumour-specific mutations in low-graded actinic keratosis. Br. J. Dermatol..

[277] Kunz M. (2014). Oncogenes in melanoma: An update. Eur. J. Cell. Biol..

[278] Hensler S., Mueller M.M. (2013). Inflammation and skin cancer: Old pals telling new stories. Cancer J..

[279] Kruk J., Duchnik E. (2014). Oxidative stress and skin diseases: Possible role of physical activity. Asian Pac. J. Cancer Prev..

[280] Mantovani A., Allavena P., Sica A., Balkwill F. (2008). Cancer-related inflammation. Nature.

[281] Munshi A., Ramesh R. (2013). Mitogen-activated protein kinases and their role in radiation response. Genes Cancer.

[282] Lopez-Camarillo C., Ocampo E.A., Casamichana M.L., Perez-Plasencia C., Alvarez-Sanchez E., Marchat L.A. (2012). Protein kinases and transcription factors activation in response to UV-radiation of skin: Implications for carcinogenesis. Int. J. Mol. Sci..

[283] Kim S.B., Kim J.E., Kang O.H., Mun S.H., Seo Y.S., Kang D.H., Yang D.W., Ryu S.Y., Lee Y.M., Kwon D.Y. (2015). Protective effect of ixerisoside A against UVB-induced pro-inflammatory cytokine production in human keratinocytes. Int. J. Mol. Med..

[284] Divya S.P., Wang X., Pratheeshkumar P., Son Y.O., Roy R.V., Kim D., Dai J., Hitron J.A., Wang L., Asha P. (2015). Blackberry extract inhibits UVB-induced oxidative damage and inflammation through MAP kinases and NF-kappaB signaling pathways in SKH-1 mice skin. Toxicol. Appl. Pharmacol..

[285] Liu Z., Shen C., Tao Y., Wang S., Wei Z., Cao Y., Wu H., Fan F., Lin C., Shan Y. (2015). Chemopreventive efficacy of menthol on carcinogen-induced cutaneous carcinoma through inhibition of inflammation and oxidative stress in mice. Food Chem. Toxicol..

[286] Foster J.G., Blunt M.D., Carter E., Ward S.G. (2012). Inhibition of PI3K signaling spurs new therapeutic opportunities in inflammatory/autoimmune diseases and hematological malignancies. Pharmacol. Rev..

[287] Davis W.J., Lehmann P.Z., Li W. (2015). Nuclear PI3K signaling in cell growth and tumorigenesis. Front. Cell Dev. Biol..

[288] Pal H.C., Athar M., Elmets C.A., Afaq F. (2015). Fisetin inhibits UVB-induced cutaneous inflammation and activation of PI3K/AKT/NFkappaB signaling pathways in SKH-1 hairless mice. Photochem. Photobiol..

[289] Weerawatanakorn M., Yang J.R., Tsai M.L., Lai C.S., Ho C.T., Pan M.H. (2014). Inhibitory effects of *Momordica grosvenori* Swingle extracts on 12-*O*-tetradecanoylphorbol 13-acetate-induced skin inflammation and tumor promotion in mouse skin. Food Funct..

[290] Lin Y., Wang F., Zhang G.L. (2014). Natural products and their derivatives regulating the janus kinase/signal transducer and activator of transcription pathway. J. Asian Nat. Prod. Res..

[291] Chun K.S., Langenbach R. (2011). The prostaglandin E2 receptor, EP2, regulates survivin expression via an EGFR/STAT3 pathway in UVB-exposed mouse skin. Mol. Carcinog..

[292] Takai E., Tsukimoto M., Harada H., Kojima S. (2011). Involvement of P2Y6 receptor in p38 MAPK-mediated COX-2 expression in response to UVB irradiation of human keratinocytes. Radiat. Res..

[293] Ivan A.L., Campanini M.Z., Martinez R.M., Ferreira V.S., Steffen V.S., Vicentini F.T., Vilela F.M., Martins F.S., Zarpelon A.C., Cunha T.M. (2014). Pyrrolidine dithiocarbamate inhibits UVB-induced skin inflammation and oxidative stress in hairless mice and exhibits antioxidant activity *in vitro*. J. Photochem. Photobiol. B.

[294] Song H., Kim J., Lee H.K., Park H.J., Nam J., Park G.B., Kim Y.S., Cho D., Hur D.Y. (2011). Selenium inhibits migration of murine melanoma cells via down-modulation of IL-18 expression. Int. Immunopharmacol..

[295] Nishio S., Yamada N., Ohyama H., Yamanegi K., Nakasho K., Hata M., Nakamura Y., Fukunaga S., Futani H., Yoshiya S. (2008). Enhanced suppression of pulmonary metastasis of malignant melanoma cells by combined administration of alpha-galactosylceramide and interleukin-18. Cancer Sci..

[296] Drexler S.K., Bonsignore L., Masin M., Tardivel A., Jackstadt R., Hermeking H., Schneider P., Gross O., Tschopp J., Yazdi A.S. (2012). Tissue-specific opposing functions of the inflammasome adaptor ASC in the regulation of epithelial skin carcinogenesis. Proc. Natl. Acad. Sci. USA.

[297] Gasparoto T.H., de Oliveira C.E., de Freitas L.T., Pinheiro C.R., Hori J.I., Garlet G.P., Cavassani K.A., Schillaci R., da Silva J.S., Zamboni D.S. (2014). Inflammasome activation is critical to the protective immune response during chemically induced squamous cell carcinoma. PLoS ONE.

[298] Yu T., Zuber J., Li J. (2015). Targeting autophagy in skin diseases. J. Mol. Med..

[299] Choi S.R., Chung B.Y., Kim S.W., Kim C.D., Yun W.J., Lee M.W., Choi J.H., Chang S.E. (2014). Activation of autophagic pathways is related to growth inhibition and senescence in cutaneous squamous cell carcinoma. Exp. Dermatol..

[300] Yoshihara N., Takagi A., Ueno T., Ikeda S. (2014). Inverse correlation between microtubule-associated protein 1A/1B-light chain 3 and p62/sequestosome-1 expression in the progression of cutaneous squamous cell carcinoma. J. Dermatol..

[301] Wright T.J., McKee C., Birch-Machin M.A., Ellis R., Armstrong J.L., Lovat P.E. (2013). Increasing the therapeutic efficacy of docetaxel for cutaneous squamous cell carcinoma through the combined inhibition of phosphatidylinositol 3-kinase/AKT signalling and autophagy. Clin. Exp. Dermatol..

[302] Serravallo M., Jagdeo J., Glick S.A., Siegel D.M., Brody N.I. (2013). Sirtuins in dermatology: Applications for future research and therapeutics. Arch. Dermatol. Res..

[303] Cao C., Lu S., Kivlin R., Wallin B., Card E., Bagdasarian A., Tamakloe T., Wang W.J., Song X., Chu W.M. (2009). SIRT1 confers protection against UVB- and H2O2-induced cell death via modulation of p53 and JNK in cultured skin keratinocytes. J. Cell. Mol. Med..

[304] Ming M., Soltani K., Shea C.R., Li X., He Y.Y. (2015). Dual role of SIRT1 in UVB-induced skin tumorigenesis. Oncogene.

[305] Ming M., Qiang L., Zhao B., He Y.Y. (2014). Mammalian SIRT2 inhibits keratin 19 expression and is a tumor suppressor in skin. Exp. Dermatol..

[306] Hida Y., Kubo Y., Murao K., Arase S. (2007). Strong expression of a longevity-related protein, SIRT1, in Bowen’s disease. Arch. Dermatol. Res..

[307] Benavente C.A., Schnell S.A., Jacobson E.L. (2012). Effects of niacin restriction on sirtuin and PARP responses to photodamage in human skin. PLoS ONE.

[308] Stahl W., Sies H. (2012). β-Carotene and other carotenoids in protection from sunlight. Am. J. Clin. Nutr..

[309] Trekli M.C., Riss G., Goralczyk R., Tyrrell R.M. (2003). Beta-carotene suppresses UVA-induced HO-1 gene expression in cultured FEK4. Free Radic. Biol. Med..

[310] Giampieri F., Alvarez-Suarez J.M., Mazzoni L., Forbes-Hernandez T.Y., Gasparrini M., Gonzàlez-Paramàs A.M., Santos-Buelga C., Quiles J.L., Bompadre S., Mezzetti B. (2014). Polyphenol-rich strawberry extract protects human dermal fibroblasts against hydrogen peroxide oxidative damage and improves mitochondrial functionality. Molecules.

[311] Horváth G., Kemény Á., Barthó L., Molnár P., Deli J., Szente L., Bozó T., Pál S., Sándor K., Szőke É. (2015). Effects of some natural carotenoids on TRPA1- and TRPV1-induced neurogenic inflammatory processes *in vivo* in the mouse skin. J. Mol. Neurosci..

[312] Lee J., Jiang S., Levine N., Watson R.R. (2000). Carotenoid supplementation reduces erythema in human skin after simulated solar radiation exposure. Proc. Soc. Exp. Biol. Med..

[313] Cho S., Lee D.H., Won C.-H., Kim S.M., Lee S., Lee M.-J., Chung J.H. (2010). Differential effects of low-dose and high-dose beta-carotene supplementation on the signs of photoaging and type I procollagen gene expression in human skin *in vivo*. Dermatology.

[314] Kune G.A., Bannerman S., Field B., Watson L.F., Cleland H., Merenstein D., Viteta L. (1992). Diet, alcohol, smoking, serum β-carotene, and vitamin A in male nonmelanocytic skin cancer patients and controls. Nutr. Cancer.

[315] Camera E., Mastrofrancesco A., Fabbri C., Daubrawa F., Picardo M., Sies H., Stahl W. (2009). Astaxanthin, canthaxanthin and beta-carotene differently affect UVA-induced oxidative damage and expression of oxidative stress-responsive enzymes. Exp. Dermatol..

[316] Suganuma K., Nakajima H., Ohtsuki M., Imokawa G. (2010). Astaxanthin attenuates the UVA-induced up-regulation of matrix-metalloproteinase-1 and skin fibroblast elastase in human dermal fibroblasts. J. Dermatol. Sci..

[317] Yoshihisa Y., Rehman M.U., Shimizu T. (2014). Astaxanthin, a xanthophyll carotenoid, inhibits ultraviolet-induced apoptosis in keratinocytes. Exp. Dermatol..

[318] Hama S., Takahashi K., Inai Y., Shiota K., Sakamoto R., Yamada A., Tsuchiya H., Kanamura K., Yamashita E., Kogure K. (2012). Protective Effects of Topical Application of a Poorly Soluble Antioxidant Astaxanthin Liposomal Formulation on Ultraviolet-Induced Skin Damage. J. Pharm. Sci..

[319] Maoka T., Tokuda H., Suzuki N., Kato H., Etoh H. (2012). Anti-oxidative, anti-tumor-promoting, and anti-carcinogensis activities of nitroastaxanthin and nitrolutein, the reaction products of astaxanthin and lutein with peroxynitrite. Mar. Drugs.

[320] Rao A.R., Sindhuja H.N., Dharmesh S.M., Sankar K.U., Sarada R., Ravishankar G.A. (2013). Effective Inhibition of skin cancer, tyrosinase, and antioxidative properties by astaxanthin and astaxanthin esters from the green alga *Haematococcus pluvialis*. J. Agric. Food Chem..

[321] Tominaga K., Hongo N., Karato M., Yamashita E. (2012). Cosmetic benefits of astaxanthin on humans subjects. Acta Biochim. Pol..

[322] Pongcharoen S., Warnnissorn P., Lertkajornsin O., Limpeanchob N. (2013). Protective effect of silk lutein on ultraviolet B-irradiated human keratinocytes. Biol. Res..

[323] Astner S., Wu A., Chen J., Philips N., Rius-Diaz F., Parrado C., Mihm M., Goukassian D., Pathak M.A., González S. (2007). Dietary lutein/zeaxanthin partially reduces photoaging and photocarcinogenesis in chronically UVB-irradiated Skh-1 hairless mice. Skin Pharmacol. Physiol..

[324] Rizwan M., Rodriguez-Blanco I., Harbottle A., Birch-Machin M.A., Watson R.E. B., Rhodes L.E. (2011). Tomato paste rich in lycopene protects against cutaneous photodamage in humans *in vivo*: A randomized controlled trial. Br. J. Dermatol..

[325] Wu N.-L., Chiang Y.-C., Huang C.-C., Fang J.-Y., Chen D.-F., Hung C.-F. (2010). Zeaxanthin inhibits PDGF-BB-induced migration in human dermal fibroblasts. Exp. Dermatol..

[326] González S., Astner S., An W., Goukassian D., Pathak M.A. (2003). Dietary lutein/zeaxanthin decreases ultraviolet B-induced epidermal hyperproliferation and acute inflammation in hairless mice. J. Investig. Dermatol..

[327] Palombo P., Fabrizi G., Ruocco V., Ruocco E., Fluhr J., Roberts R., Morganti P. (2007). Beneficial Long-Term Effects of Combined Oral/Topical Antioxidant treatment with the carotenoids lutein and zeaxanthin on human skin: A double-blind, placebo-controlled study. Skin Pharmacol. Physiol..

[328] Heo S.-J., Jeon Y.-J. (2009). Protective effect of fucoxanthin isolated from *Sargassum siliquastrum* on UV-B induced cell damage. J. Photochem. Photobiol. B.

[329] Article O., Zheng J., Piao M.J., Keum Y.S., Kim H.S., Hyun J.W. (2013). Fucoxanthin protects cultured human keratinocytes against oxidative stress by blocking free radicals and inhibiting apoptosis. Biomol. Ther..

[330] Zheng J., Piao M.J., Kim K.C., Yao C.W., Cha J.W., Hyun J.W. (2014). Fucoxanthin enhances the level of reduced glutathione via the Nrf2-mediated pathway in human keratinocytes. Mar. Drugs.

[331] Urikura I., Sugawara T., Hirata T. (2011). Protective effect of fucoxanthin against UVB-induced skin photoaging in hairless mice. Biosci. Biotechnol. Biochem..

[332] Shimoda H., Tanaka J., Shan S.-J., Maoka T. (2010). Anti-pigmentary activity of fucoxanthin and its influence on skin mRNA expression of melanogenic molecules. J. Pharm. Pharmacol..

[333] Feingold K.R. (2007). Thematic review series: Skin lipids. The role of epidermal lipids in cutaneous permeability barrier homeostasis. J. Lipid. Res..

[334] McDaniel J.C., Massey K., Nicolaou A. (2011). Fish oil supplementation alters levels of lipid mediators of inflammation in microenvironment of acute human wounds. Wound Repair Regen..

[335] Barcelos R.C., de Mello-Sampayo C., Antoniazzi C.T., Segat H.J., Silva H., Veit J.C., Piccolo J., Emanuelli T., Bürger M.E., Silva-Lima B. (2015). Oral supplementation with fish oil reduces dryness and pruritus in the acetone-induced dry skin rat model. J Dermatol Sci..

[336] McCusker M.M., Grant-Kels J.M. (2010). Healing fats of the skin: The structural and immunologic roles of the omega-6 and omega-3 fatty acids. Clin. Dermatol..

[337] Kim W., Khan N.A., McMurray D.N., Prior I.A., Wang N., Chapkin R.S. (2010). Regulatory activity of polyunsaturated fatty acids in T-cell signaling. Prog. Lipid. Res..

[338] Storey A., McArdle F., Friedmann P.S., Jackson M.J., Rhodes L.E. (2005). Eicosapentaenoic acid and docosahexaenoic acid reduce UVB- and TNF-alpha-induced IL-8 secretion in keratinocytes and UVB-induced IL-8 in fibroblasts. J. Investig. Dermatol..

[339] Cansell M.S., Moussaoui N., Mancini M. (2007). Prostaglandin E2 and interleukin-8 production in human epidermal keratinocytes exposed to marine lipid-based liposomes. Int. J. Pharm..

[340] Chene G., Dubourdeau M., Balard P., Escoubet-Lozach L., Orfila C., Berry A., Bernad J., Aries M.F., Charveron M., Pipy B. (2007). *n*-3 and *n*-6 polyunsaturated fatty acids induce the expression of COX-2 via PPARgamma activation in human keratinocyte HaCaT cells. Biochim. Biophys. Acta.

[341] Obata T., Nagakura T., Masaki T., Maekawa K., Yamashita K. (1999). Eicosapentaenoic acid inhibits prostaglandin D2 generation by inhibiting cyclo-oxygenase-2 in cultured human mast cells. Clin. Exp. Allergy.

[342] Vanderveen E.E., Grekin R.C., Swanson N.A., Kragballe K. (1986). Arachidonic acid metabolites in cutaneous carcinomas. Evidence suggesting that elevated levels of prostaglandins in basal cell carcinomas are associated with an aggressive growth pattern. Arch. Dermatol..

[343] Fischer M.A., Black H.S. (1991). Modification of membrane composition, eicosanoid metabolism, and immunoresponsiveness by dietary omega-3 and omega-6 fatty acid sources, modulators of ultraviolet-carcinogenesis. Photochem. Photobiol..

[344] Kim H.H., Shin C.M., Park C.H., Kim K.H., Cho K.H., Eun H.C., Chung J.H. (2005). Eicosapentaenoic acid inhibits UV-induced MMP-1 expression in human dermal fibroblasts. J Lipid. Res..

[345] Jeon B.K., Kang M.K., Lee G.T., Lee K.K., Lee H.S., Woo W.H., Mun Y.J. (2014). EPA attenuates ultraviolet radiation-induced downregulation of aquaporin-3 in human keratinocytes. Arch. Pharm. Res..

[346] Jin X.J., Kim E.J., Oh I.K., Kim Y.K., Park C.H., Chung J.H. (2010). Prevention of UV-induced skin damages by 11,14,17-eicosatrienoic acid in hairless mice *in vivo*. J. Korean Med. Sci..

[347] Campelo A.P., Campelo M.W., Brito G.A., Jamacaru F.V., Leitao R.F., Vasconcelos P.R. (2015). Oil mixes omega 9, 6 and 3, enriched with seaweed, promoted reduction of thermal burned modulating NF-kB and Ki-67. Acta Cir. Bras..

[348] Black H.S., Thornby J.I., Gerguis J., Lenger W. (1992). Influence of dietary omega-6, -3 fatty acid sources on the initiation and promotion stages of photocarcinogenesis. Photochem Photobiol..

[349] Lou Y.R., Peng Q.Y., Li T., Medvecky C.M., Lin Y., Shih W.J., Conney A.H., Shapses S., Wagner G.C., Lu Y.P. (2011). Effects of high-fat diets rich in either omega-3 or omega-6 fatty acids on UVB-induced skin carcinogenesis in SKH-1 mice. Carcinogenesis.

[350] Shahbakhti H., Watson R.E., Azurdia R.M., Ferreira C.Z., Garmyn M., Rhodes L.E. (2004). Influence of eicosapentaenoic acid, an omega-3 fatty acid, on ultraviolet-B generation of prostaglandin-E2 and proinflammatory cytokines interleukin-1 beta, tumor necrosis factor-alpha, interleukin-6 and interleukin-8 in human skin *in vivo*. Photochem. Photobiol..

[351] Rhodes L.E., Shahbakhti H., Azurdia R.M., Moison R.M., Steenwinkel M.J., Homburg M.I., Dean M.P., McArdle F., van Henegouwen G.M.J.B., Epe B. (2003). Effect of eicosapentaenoic acid, an omega-3 polyunsaturated fatty acid, on UVR-related cancer risk in humans. An assessment of early genotoxic markers. Carcinogenesis.

[352] Pilkington S.M., Massey K.A., Bennett S.P., Al-Aasswad N.M., Roshdy K., Gibbs N.K., Friedmann P.S., Nicolaou A., Rhodes L.E. (2013). Randomized controlled trial of oral omega-3 PUFA in solar-simulated radiation-induced suppression of human cutaneous immune responses. Am. J. Clin. Nutr..

[353] Pilkington S.M., Rhodes L.E., Al-Aasswad N.M., Massey K.A., Nicolaou A. (2014). Impact of EPA ingestion on COX- and LOX-mediated eicosanoid synthesis in skin with and without a pro-inflammatory UVR challenge—Report of a randomised controlled study in humans. Mol Nutr. Food. Res..

[354] Wallingford S.C., Hughes M.C., Green A.C., van der Pols J.C. (2013). Plasma omega-3 and omega-6 concentrations and risk of cutaneous basal and squamous cell carcinomas in Australian adults. Cancer Epidemiol. Biomarkers Prev..

[355] Van Dam R.M., Huang Z., Giovannucci E., Rimm E.B., Hunter D.J., Colditz G.A., Stampfer M.J., Willett W.C. (2000). Diet and basal cell carcinoma of the skin in a prospective cohort of men. Am. J. Clin. Nutr..

[356] Matsumoto Y., Sahara H., Fujita T., Hanashima S., Yamazaki T., Takahashi S., Sugawara F., Mizushina Y., Ohta K., Takahashi N. (2000). A novel immunosuppressive agent, SQDG, derived from sea urchin. Transplant. Proc..

[357] Bruno A., Rossi C., Marcolongo G., di Lena A., Venzo A., Berrie C.P., Corda D. (2005). Selective *in vivo* anti-inflammatory action of the galactolipid monogalactosyldiacylglycerol. Eur. J. Pharmacol..

[358] Colombo D., Tringali C., Franchini L., Cirillo F., Venerando B. (2011). Glycoglycerolipid analogues inhibit PKC translocation to the plasma membrane and downstream signaling pathways in PMA-treated fibroblasts and human glioblastoma cells, U87MG. Eur. J. Med. Chem..

[359] Colombo D., Compostella F., Ronchetti F., Scala A., Toma L., Kuchide M., Tokuda H., Nishino H. (2000). Anti-tumor-promoting effects of glycoglycerolipid analogues on two-stage mouse skin carcinogenesis. Cancer Lett..

[360] McCarty M.F., O’Keefe J.H., DiNicolantonio J.J. (2015). Carvedilol and spirulina may provide important health protection to smokers and other nicotine addicts: A call for pertinent research. Mol. Med..

[361] Yang F., Wong K.H., Yang Y., Li X., Jiang J., Zheng W., Wu H., Chen T. (2014). Purification and *in vitro* antioxidant activities of tellurium-containing phycobiliproteins from tellurium-enriched *Spirulina platensis*. Drug. Des. Devel. Ther..

[362] Gupta N.K., Gupta K.P. (2012). Effects of C-Phycocyanin on the representative genes of tumor development in mouse skin exposed to 12-*O*-tetradecanoyl-phorbol-13-acetate. Environ. Toxicol. Pharmacol..

[363] Bandaranayake W.M. (1998). Mycosporines: Are they nature’s sunscreens?. Nat. Prod. Rep..

[364] Carreto J.I., Carignan M.O. (2011). Mycosporine-like amino acids: Relevant secondary metabolites. Chemical and ecological aspects. Mar. Drugs.

[365] Sinha R.P., Singh S.P., Hader D.P. (2007). Database on mycosporines and mycosporine-like amino acids (MAAs) in fungi, cyanobacteria, macroalgae, phytoplankton and animals. J. Photochem. Photobiol. B.

[366] Llewellyn C.A., Airs R.L. (2010). Distribution and abundance of MAAs in 33 species of microalgae across 13 classes. Mar. Drugs.

[367] Conde F.R., Churio M.S., Previtali C.M. (2004). The deactivation pathways of the excited-states of the mycosporine-like amino acids shinorine and porphyra-334 in aqueous solution. Photochem. Photobiol. Sci..

[368] Suh H.J., Lee H.W., Jung J. (2003). Mycosporine glycine protects biological systems against photodynamic damage by quenching singlet oxygen with a high efficiency. Photochem. Photobiol..

[369] Yakovleva I., Bhagooli R., Takemura A., Hidaka M. (2004). Differential susceptibility to oxidative stress of two scleractinian corals: Antioxidant functioning of mycosporine-glycine. Comp. Biochem. Physiol. B Biochem. Mol. Biol..

[370] Zhang L., Li L., Wu Q. (2007). Protective effects of mycosporine-like amino acids of *Synechocysti*s sp. PCC 6803 and their partial characterization. J. Photochem. Photobiol. B.

[371] Ryu J., Park S.J., Kim I.H., Choi Y.H., Nam T.J. (2014). Protective effect of porphyra-334 on UVA-induced photoaging in human skin fibroblasts. Int. J. Mol. Med..

[372] Suh S.S., Hwang J., Park M., Seo H.H., Kim H.S., Lee J.H., Moh S.H., Lee T.K. (2014). Anti-inflammation activities of mycosporine-like amino acids (MAAs) in response to UV radiation suggest potential anti-skin aging activity. Mar. Drugs.

[373] Thomas N.V., Kim S.K. (2013). Beneficial effects of marine algal compounds in cosmeceuticals. Mar. Drugs.

[374] Chinembiri T.N., du Plessis L.H., Gerber M., Hamman J.H., du Plessis J. (2014). Review of natural compounds for potential skin cancer treatment. Molecules.

[375] Hwang H., Chen T., Nines R.G., Shin H.C., Stoner G.D. (2006). Photochemoprevention of UVB-induced skin carcinogenesis in SKH-1 mice by brown algae polyphenols. Int. J. Cancer.

[376] Kim S., You D.H., Han T., Choi E.M. (2014). Modulation of viability and apoptosis of UVB-exposed human keratinocyte HaCaT cells by aqueous methanol extract of laver (*Porphyra yezoensis*). J. Photochem. Photobiol. B.

[377] Heo S.J., Ko S.C., Cha S.H., Kang D.H., Park H.S., Choi Y.U., Kim D., Jung W.K., Jeon Y.J. (2009). Effect of phlorotannins isolated from *Ecklonia cava* on melanogenesis and their protective effect against photo-oxidative stress induced by UV-B radiation. Toxicol. In Vitro.

[378] Goh L.P., Loh S.P., Fatimah M.Y., Perumal K. (2009). Bioaccessibility of Carotenoids and Tocopherols in Marine Microalgae, *Nannochloropsis* sp. and *Chaetoceros* sp. Malays J. Nutr..

[379] Carballo-Cardenas E.C., Tuan P.M., Janssen M., Wijffels R.H. (2003). Vitamin E (alpha-tocopherol) production by the marine microalgae *Dunaliella tertiolecta* and *Tetraselmis suecica* in batch cultivation. Biomol. Eng..

[380] Pal A., Alam S., Singhal J., Kumar R., Ansari K.M., Das M. (2013). Protective effect of topical application of alpha-tocopherol and/or *N*-acetyl cysteine on argemone oil/alkaloid-induced skin tumorigenesis in mice. Nutr. Cancer.

[381] Jiang Q., Elson-Schwab I., Courtemanche C., Ames B.N. (2000). Gamma-tocopherol and its major metabolite, in contrast to alpha-tocopherol, inhibit cyclooxygenase activity in macrophages and epithelial cells. Proc. Natl. Acad. Sci. USA.

[382] Jiang Q., Ames B.N. (2003). Gamma-tocopherol, but not alpha-tocopherol, decreases proinflammatory eicosanoids and inflammation damage in rats. FASEB J..

[383] Jiang Q., Lykkesfeldt J., Shigenaga M.K., Shigeno E.T., Christen S., Ames B.N. (2002). Gamma-tocopherol supplementation inhibits protein nitration and ascorbate oxidation in rats with inflammation. Free Radic Biol. Med..

[384] Yoshida E., Watanabe T., Takata J., Yamazaki A., Karube Y., Kobayashi S. (2006). Topical application of a novel, hydrophilic gamma-tocopherol derivative reduces photo-inflammation in mice skin. J. Investig. Dermatol..

[385] Trevithick J.R., Xiong H., Lee S., Shum D.T., Sanford S.E., Karlik S.J., Norley C., Dilworth G.R. (1992). Topical tocopherol acetate reduces post-UVB, sunburn-associated erythema, edema, and skin sensitivity in hairless mice. Arch. Biochem. Biophys..

[386] Rahman S., Bhatia K., Khan A.Q., Kaur M., Ahmad F., Rashid H., Athar M., Islam F., Raisuddin S. (2008). Topically applied vitamin E prevents massive cutaneous inflammatory and oxidative stress responses induced by double application of 12-*O*-tetradecanoylphorbol-13-acetate (TPA) in mice. Chem. Biol. Interact..

[387] Gensler H.L., Magdaleno M. (1991). Topical vitamin E inhibition of immunosuppression and tumorigenesis induced by ultraviolet irradiation. Nutr. Cancer.

[388] Brown M., Miller K. (1992). The ascorbic acid content of eleven species of microalgae used in mariculture. J. Appl. Phycol..

[389] Maia Campos P.M., Gianeti M.D., Camargo F.B., Gaspar L.R. (2012). Application of tetra-isopalmitoyl ascorbic acid in cosmetic formulations: Stability studies and *in vivo* efficacy. Eur. J. Pharm. Biopharm..

[390] Gehin A., Guyon C., Nicod L. (2006). Glyphosate-induced antioxidant imbalance in HaCaT: The protective effect of Vitamins C and E. Environ. Toxicol. Pharmacol..

[391] Lin J.R., Qin H.H., Wu W.Y., He S.J., Xu J.H. (2014). Vitamin C protects against UV irradiation-induced apoptosis through reactivating silenced tumor suppressor genes p21 and p16 in a Tet-dependent DNA demethylation manner in human skin cancer cells. Cancer Biother. Radiopharm..

[392] Besaratinia A., Kim S.I., Bates S.E., Pfeifer G.P. (2007). Riboflavin activated by ultraviolet A1 irradiation induces oxidative DNA damage-mediated mutations inhibited by vitamin C. Proc. Natl. Acad. Sci. USA.

[393] Plaza M., Santoyo S., Jaime L., García-Blairsy Reina G., Herrero M., Señoráns F.J., Ibáñez E. (2010). Screening for bioactive compounds from algae. J. Pharm. Biomed. Anal..

[394] Khanavi M., Gheidarloo R., Sadati N., Ardekani M.R., Nabavi S.M., Tavajohi S., Ostad S.N. (2012). Cytotoxicity of fucosterol containing fraction of marine algae against breast and colon carcinoma cell line. Pharmacogn. Mag..

[395] Kim M.S., Oh G.H., Kim M.J., Hwang J.K. (2013). Fucosterol inhibits matrix metalloproteinase expression and promotes type-1 procollagen production in UVB-induced HaCaT cells. Photochem. Photobiol..

[396] Hwang E., Park S.Y., Sun Z.W., Shin H.S., Lee D.G., Yi T.H. (2014). The protective effects of fucosterol against skin damage in UVB-irradiated human dermal fibroblasts. Mar. Biotechnol..

